# Proteostatic Regulation of MEP and Shikimate Pathways by Redox-Activated Photosynthesis Signaling in Plants Exposed to Small Fungal Volatiles

**DOI:** 10.3389/fpls.2021.637976

**Published:** 2021-03-05

**Authors:** Kinia Ameztoy, Ángela María Sánchez-López, Francisco José Muñoz, Abdellatif Bahaji, Goizeder Almagro, Edurne Baroja-Fernández, Samuel Gámez-Arcas, Nuria De Diego, Karel Doležal, Ondřej Novák, Ales Pěnčík, Adán Alpízar, Manuel Rodríguez-Concepción, Javier Pozueta-Romero

**Affiliations:** ^1^Instituto de Agrobiotecnología (Consejo Superior de Investigaciones Científicas/Gobierno de Navarra), Mutilva, Spain; ^2^Department of Chemical Biology and Genetics, Centre of the Region Haná for Biotechnological and Agricultural Research, Faculty of Science, Palacký University, Olomouc, Czechia; ^3^Laboratory of Growth Regulators, Faculty of Science of Palackı University and Institute of Experimental Botany of the Czech Academy of Sciences, Olomouc, Czechia; ^4^Unidad de Proteómica Centro Nacional de Biotecnología, Consejo Superior de Investigaciones Científicas, Madrid, Spain; ^5^Centre for Research in Agricultural Genomics (CRAG) CSIC-IRTA-UAB-UB, Barcelona, Spain; ^6^Instituto de Hortofruticultura Subtropical y Mediterránea “La Mayora” (IHSM-UMA-CSIC) Campus de Teatinos, Málaga, Spain

**Keywords:** : Clp protease system, MEP pathway, plant–microbe interaction, proteostatic regulation, PQC system, redox regulation, chloroplast-to-nucleus retrograde signaling

## Abstract

Microorganisms produce volatile compounds (VCs) with molecular masses of less than 300 Da that promote plant growth and photosynthesis. Recently, we have shown that small VCs of less than 45 Da other than CO_2_ are major determinants of plant responses to fungal volatile emissions. However, the regulatory mechanisms involved in the plants’ responses to small microbial VCs remain unclear. In *Arabidopsis thaliana* plants exposed to small fungal VCs, growth promotion is accompanied by reduction of the thiol redox of Calvin-Benson cycle (CBC) enzymes and changes in the levels of shikimate and 2-C-methyl-D-erythritol 4-phosphate (MEP) pathway-related compounds. We hypothesized that plants’ responses to small microbial VCs involve post-translational modulation of enzymes of the MEP and shikimate pathways via mechanisms involving redox-activated photosynthesis signaling. To test this hypothesis, we compared the responses of wild-type (WT) plants and a *cfbp1* mutant defective in a redox-regulated isoform of the CBC enzyme fructose-1,6-bisphosphatase to small VCs emitted by the fungal phytopathogen *Alternaria alternata.* Fungal VC-promoted growth and photosynthesis, as well as metabolic and proteomic changes, were substantially weaker in *cfbp1* plants than in WT plants. In WT plants, but not in *cfbp1* plants, small fungal VCs reduced the levels of both transcripts and proteins of the stromal Clp protease system and enhanced those of plastidial chaperonins and co-chaperonins. Consistently, small fungal VCs promoted the accumulation of putative Clp protease clients including MEP and shikimate pathway enzymes. *clpr1-2* and *clpc1* mutants with disrupted plastidial protein homeostasis responded weakly to small fungal VCs, strongly indicating that plant responses to microbial volatile emissions require a finely regulated plastidial protein quality control system. Our findings provide strong evidence that plant responses to fungal VCs involve chloroplast-to-nucleus retrograde signaling of redox-activated photosynthesis leading to proteostatic regulation of the MEP and shikimate pathways.

## One Sentence Summary

We provide strong evidence that plant responses to volatile compounds emitted by the fungal phytopathogen *Alternaria alternata* involve chloroplast-to-nucleus retrograde signaling of redox-activated photosynthesis leading to proteostatic regulation of the MEP and shikimate pathways.

## Introduction

Phylogenetically diverse microorganisms (including plant pathogens) can produce volatile compounds (VCs) with molecular masses of less than 300 Da that promote plant growth, photosynthesis, and developmental changes (Ryu et al., 2003; Zhang et al., 2008; Ditengou et al., 2015; Garnica-Vergara et al., 2016; Sánchez-López et al., 2016b; García-Gómez et al., 2019; Guo et al., 2020). Recently, we have shown that small VCs of less than 45 Da other than CO_2_ are major determinants of plant responses to microbial volatiles (García-Gómez et al., 2019). In Arabidopsis, promotion of growth and photosynthesis by small VCs emitted by the fungal phytopathogen *Alternaria alternata* is associated with enhanced levels of soluble sugars, starch formed by the activation of non-canonical biosynthetic pathway(s), anthocyanins, and photosynthetic pigments (Sánchez-López et al., 2016b; Ameztoy et al., 2019; García-Gómez et al., 2019). This response is also associated with reductions in the content of abscisic acid (ABA) in the leaves together with increased levels of cytokinins (CKs) derived from the plastid-localized 2-C-methyl-D-erythritol 4-phosphate (MEP) pathway (Sánchez-López et al., 2016b; Ameztoy et al., 2019). Transcriptomic changes induced by small microbial VCs are similar to those occurring in plants cultured under conditions that enhance photosynthesis such as exposure to elevated CO_2_ and increased irradiance (García-Gómez et al., 2019), despite substantial differences in the induced developmental and metabolic changes (Sánchez-López et al., 2016b; García-Gómez et al., 2019). This suggests that (i) transcriptional changes in microbial VC-exposed plants are mainly due to enhanced photosynthesis signaling, and (ii) regulation of some plant responses to small fungal VCs is primarily non-transcriptional (García-Gómez et al., 2019). This hypothesis is supported by recent quantitative and site-specific redox-proteomic analyses showing that small microbial VCs promote global thiol redox proteome changes including the reduction of highly conserved Cys residues in redox-regulated Calvin-Benson cycle (CBC) enzymes [e.g., phosphoribulokinase, sedoheptulose-1,7-bisphosphatase, and one of the two isoforms of plastid-localized fructose-1,6-bisphosphatase (cFBP1)], as well as proteins involved in the photochemical reactions of photosynthesis (Ameztoy et al., 2019).

The carbon fixed by the CBC can be used for sucrose and starch biosynthesis, and is also an essential source of substrates for the synthesis of secondary metabolites important for growth and development (e.g., phenylpropanoids and isoprenoids) via the plastid-localized shikimate and MEP pathways. Modulation of the metabolic flux toward the shikimate pathway involves the provision of substrates (e.g., erythrose-4-phosphate, E4P) by the CBC (Henkes et al., 2001), together with feedback allosteric inhibition and redox regulation of the pathway’s first enzyme, phospho-2-dehydro-3-deoxyheptonate aldolase (DHS) (Entus et al., 2002; Tzin et al., 2012). DHS is recognized by the Clp protease system, and it was suggested that Clp-regulated proteolysis might provide a mechanism of control of the shikimate pathway activity (Nishimura et al., 2013). The MEP pathway uses pyruvate and glyceraldehyde 3-phosphate (GAP) to produce the universal prenyl diphosphate precursors of isoprenoid compounds. Studies on the mechanisms controlling the metabolic flux through the MEP pathway in leaves have revealed multiple levels of regulation. One level is based on the dependence of the MEP pathway on the availability of GAP, which in turn depends on photosynthetic activity (Ghirardo et al., 2010; Pokhilko et al., 2015). Another level involves coarse control by transcriptional regulation of the expression of MEP pathway genes (Carretero-Paulet et al., 2002; Córdoba et al., 2009; Vranová et al., 2013) while a third finer level of regulation is based on post-translational control of the first enzymatic step of the MEP pathway, which is catalyzed by 1-deoxy-D-xylulose-5-phosphate synthase (DXS). In keeping with its major contribution to the control of the MEP pathway flux (Wright et al., 2014), the abundance and activity of DXS are regulated by post-translational mechanisms including (i) degradation or activation through a pathway involving the J-protein adaptor J20, Hsp70 chaperones, the Hsp100 chaperones ClpB3 and ClpC1 and the Clp protease complex (Pulido et al., 2013; Llamas et al., 2017; Rodríguez-Concepción et al., 2019), and (ii) allosteric inhibition by MEP pathway products (Banerjee et al., 2013; Ghirardo et al., 2014).

Fungal VC-promoted accumulation of chlorophylls and plastidial CKs in leaves is not associated with enhanced expression of genes encoding enzymes directly involved in the synthesis of these compounds (Sánchez-López et al., 2016b). Because the MEP pathway flux depends strongly on photosynthetically produced GAP, this indicates that enhanced MEP pathway-derived metabolism in response to microbial VCs may be at least partly due to enhanced photosynthetic activity (García-Gómez et al., 2019). Alternatively, and/or additionally, enhancement of MEP pathway-derived metabolism by microbial VCs could be caused by non-transcriptional up-regulation of the expression of MEP pathway enzymes induced by signaling of redox-activated photosynthesis. To test these hypotheses and clarify the mechanisms involved in plant responses to microbial VCs, we compared the proteomic, metabolic, and hormonal responses of wild-type (WT) Arabidopsis plants and a cFBP1 knockout mutant (*cfbp1*) to VCs emitted by *A. alternata.* Because small VCs of less than 45 Da are major determinants of plant responses to fungal volatile emissions (García-Gómez et al., 2019), here we characterized the responses of plants to fungal VCs filtered by a charcoal type that adsorbs volatile organic compounds (VOCs) of molecular masses higher than 45 Da. Arabidopsis expresses two plastidial fructose-1,6-bisphosphatases: cFBP1, which is redox-regulated by light, and cFBP2, which is not redox-regulated (Serrato et al., 2009; Rojas-González et al., 2015). Because the CBC is subject to redox control (Michelet et al., 2013) and appears to be redox-activated by microbial VCs (Ameztoy et al., 2019), we reasoned that if the microbial VC-promoted response is controlled by substrate provision and/or non-transcriptional regulation of CBC-related pathways by enhanced photosynthesis, the response of *cfbp1* plants to small fungal VCs should be weaker than that of WT plants. Conversely, if redox activation of photosynthesis does not play an important role in regulating CBC-related pathways influencing growth and development, the responses of WT and *cfbp1* plants to microbial VCs should be similar. The results presented here are consistent with the former scenario and provide evidence that plant responses to small fungal VCs are subject to post-translational regulation of the homeostasis of MEP and shikimate pathway enzymes by the plastid protein quality control (PQC) system in which chloroplast-to-nucleus retrograde signaling of redox-activated photosynthesis plays an important role. To our knowledge, this work provides the first example of an environmentally triggered modulation of the expression of genes encoding subunits of the Clp protease complex as a way to change the levels of MEP and shikimate pathway enzymes and hence regulate chloroplast metabolism. Therefore, our results also show that investigating plant responses to microbial VCs is an excellent way of unveiling fundamental mechanisms involved not only in plant–microbe interactions but also in metabolic regulation in plants.

## Materials and Methods

### Plant and Microbial Cultures, Growth Conditions, and Sampling

The work was carried out using *Arabidopsis thaliana* L. (Heynh) ecotype Columbia (Col-0), the *cfbp1* knockout mutant, line GK-472G06-019879 (At3g54050) (Rojas-González et al., 2015), the *cfbp2* knockout mutant (SALK_053799) (At5g64380), and the *clpc1* (SALK_014058) (At5g50920) and *clpr1-2* (SALK_088407) (At1g49970) knockout mutants (Pulido et al., 2016). The knockout status of *cfbp2* was confirmed by RT-PCR using specific primers of the region downstream of the T-DNA insertion site. To this end, total RNA was extracted from leaves using the trizol method according to the manufacturer’s procedure (Invitrogen). RNA was treated with RNase-free DNase (Takara). RT-PCR was conducted with Super-Script III one-step RT-PCR with Platinum Taq DNA polymerase kit (12574-018; Invitrogen) using 100 ng of RNA and the 5′-AACCCTTTGGCTTTTCTCGT-3′ and 5′-GAGGCAATCTTTGGTGAAGC-3′ *cFBP2* specific primers. EF-1 alpha was used as a loading control. PCR products were separated on 1% (w/v) agarose gels containing ethidium bromide and visualized by ultraviolet light. Seeds were sown and plants were cultured in Petri dishes (92 × 16 mm, Sarstedt, Ref. 82.1472.001) containing half-strength agar-solidified Murashige and Skoog (MS) (Phytotechlab M519) medium in growth chambers providing “long day” 16-h light (90 μmol photons s^–1^ m^–2^), 22°C/8 h dark, 18°C cycles. *A. alternata* was cultured in Petri dishes containing agar-solidified MS medium supplemented with 90 mM sucrose. Effects of small, fungal VCs on plants were investigated using the “box-in-box” co-cultivation system described in García-Gómez et al. (2019) in which plants are grown in the vicinity of fungal cultures covered with charcoal filters that adsorb VOCs of molecular masses higher than 45 Da. VC treatment started at 14 days after the sowing growth stage of plants. At the indicated incubation periods, leaves were harvested, immediately freeze-clamped, and ground to a fine powder in liquid nitrogen with a pestle and mortar.

### Determination of Gas Exchange Rates and Photosynthetic Parameters

Gas exchange rates were determined as described by Sánchez-López et al. (2016b) using a LI-COR 6400 gas exchange portable photosynthesis system (LI-COR, Lincoln, NE, United States). The net rate of CO_2_ assimilation (*A*_n_) was calculated as described by von Caemmerer and Farquhar (1981). The maximum rate of carboxylation by Rubisco (*V*_cmax_), the maximum electron transport demand for RuBP regeneration (*J*_max_), and the triose phosphate use (TPU) values were calculated from *A_n_/*intracellular CO_2_ concentration (*C*_i_) curves according to Long and Bernacchi (2003). To avoid miscalculation of *A*_n_ and *C*_i_ because of leakage into the gasket of the gas analyzer, we performed CO_2_ response curves using an empty chamber. The values obtained for *A*_n_ and *C*_i_ in the empty chamber were compared with those of the chamber filled with a leaf and subtracted from the values obtained with the empty chamber. The rate of mitochondrial respiration in the dark necessary for TPU calculation was determined by measuring the rate of CO_2_ evolution in the dark.

### Analytical Procedures

Tocopherols and flavonols (e.g., quercetin and kaempferol) were measured according to Yin et al. (2012). Levels of CKs and ABA were determined following Novák et al. (2008) and Floková et al. (2014), respectively. Indole acetic acid (IAA) and IAA conjugate contents were determined essentially as described by Pěnčík et al. (2009). Recovery experiments were carried out by adding known amounts of metabolite standards to the frozen slurry immediately after the addition of extraction solutions. The difference between the measurements from samples with and without added standards was used as an estimate of the percentage recovery. All presented concentrations of these metabolites were corrected for losses during extraction. Total photosynthetic pigments and anthocyanin contents were quantified according to Lichtenthaler (1987) and Teow et al. (2007), respectively.

### Real-Time Quantitative PCR

Total RNA was extracted from frozen Arabidopsis leaves of *in vitro* cultured plants using the Trizol method, following treatment with RNase-free DNase. RNA (1.5 μg) was reverse-transcribed using polyT primers and an Expand Reverse Transcriptase kit (Roche) according to the manufacturer’s instructions. RT-PCR amplification was performed as described by Sánchez-López et al. (2016b) using primers listed in Supplementary Table 1, and their specificity was checked by separating the obtained products on 1.8% agarose gels. The specificity of the PCR amplifications was checked by acquiring heat dissociation curves (from 60 to 95°C). Comparative threshold values were normalized to EF-1 alfa internal control.

### Label-Free Proteomic Analysis

Protein samples were prepared as described in Sánchez-López et al. (2016a), but the TMT-6 Plex labeling step was omitted. Briefly, 200 mg of leaf material was ground into a fine powder under liquid nitrogen using a precooled mortar and pestle. The powder was then mixed with chaotropic lysis buffer containing 8.4 M urea (USB Corporation, Cleveland, OH), 2.4 M thiourea (Sigma-Aldrich), 5% CHAPS (Sigma-Aldrich), 5 mM TCEP (Sigma-Aldrich), and a protease inhibitor cocktail (Sigma-Aldrich), and incubated for 15 min on ice. Homogenization of the pellet was achieved by ultrasonication for 5 min on ultrasonic bath Branson 2510 (Marshall Scientific, New Hampshire, United States). The homogenate was centrifuged at 20,000×*g* for 10 min at 4°C, and the supernatant containing the solubilized proteins was used for further analysis. Samples were then precipitated by methanol/chloroform method and re-suspended in 100 μl of multichaotropic sample solution UTT buffer [7 M Urea, 2 M thiourea, 100 mM TEAB (Sigma-Aldrich)]. Twenty micrograms of sample quantified by colorimetric method Pearse was reduced with 2 μl of 50 mM TCEP, pH 8.0, at 37°C for 60 min, followed by addition of 1 μl of 200 mM cysteine-blocking reagent MMTS (SCIEX, Foster City, CA) for 10 min at room temperature. Sample was diluted to 100 μl to reduce the urea concentration with 25 mM TEAB. Finally, digestion was initiated by adding 1 μg of Pierce MS-grade trypsin (Thermo-Fisher Scientific Inc.) to each sample in a ratio 1:20 (w/w), and then incubated at 37°C overnight on a shaker. Sample digestion was evaporated to dryness in a vacuum concentrator. The liquid chromatography and mass spectrometer analysis was carried out essentially as described in Sánchez-López et al. (2016a) but with minimal modifications. Digested sample was desalted using Stage-Tips with Empore 3M C18 disks (Sigma-Aldrich), and peptides were separated using a 250 min gradient ranging from 2 to 90% mobile phase B (mobile phase A: 2% acetonitrile, 0.1% formic acid; mobile phase B: 100% acetonitrile, 0.1% formic acid). Injection volume was 5 μl. All data were acquired using information-dependent acquisition mode with Analyst TF 1.7 software (SCIEX, United States). Data analysis and quantification were conducted as described in Sánchez-López et al. (2016a) using software from Proteobotics (Madrid, Spain). Differential regulation was measured using linear models, and statistical significance was measured using *q* values (FDR). All analyses were conducted using software from Proteobotics (Madrid, Spain). The cutoff for differentially regulated proteins was established at a FDR ≤ 0.05% and log2 ratios (+VCs treatment vs. -VCs treatment) of either >0.35 (for proteins whose expression is up-regulated by VCs) and <-0.35 (for proteins whose expression is down-regulated by VCs).

### Statistical Analysis

Unless otherwise indicated, presented data are means (±SE) obtained from three to four independent experiments, with three to five replicates for each experiment. The significance of differences between plants exposed and not exposed to *A. alternata* VCs was statistically evaluated with Student’s *t*-test using the SPSS software. Differences were considered significant if *P* < 0.05.

## Results

### Small Fungal VCs Do Not Stimulate Growth of *cfbp1* Plants

**FIGURE 1:**
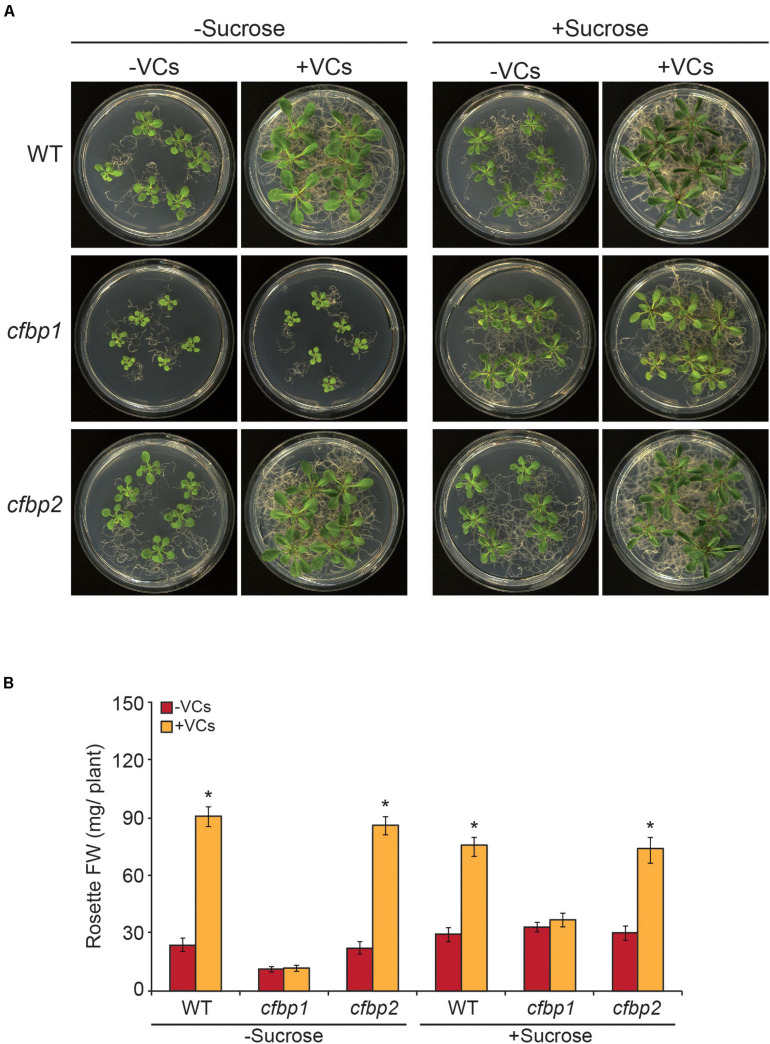
Fungal VCs do not promote growth of *cfbp1* plants. **(A)** External phenotypes and **(B)** rosette FW of WT, *cfbp1*, and *cfbp2* plants cultured in the absence or continuous presence of small volatile compounds (VCs) released by adjacent *Alternaria alternata* cultures for 1 week. Values in “B” are means ± SE for three biological replicates (each comprising a pool of 12 plants) from four independent experiments. Asterisks indicate significant differences relative to plants not cultured with small VCs released by adjacent fungal cultures based on Student’s *t*-test (*P* < 0.05).

We compared growth responses of *in vitro* cultured WT, *cfbp1*, and *cfbp2* plants to small VCs emitted by adjacent *A. alternata* cultures. In the absence of fungal VCs, shoots of the *cfbp2* mutant displayed a WT growth phenotype (Figure 1). In keeping with Rojas-González et al. (2015), *cfbp1* plants were smaller than WT plants, a phenotype that could be rescued by sucrose supplementation (Figure 1). As in WT plants, fungal VCs promoted growth of *cfbp2* plants cultured in MS with or without sucrose supplementation (Figure 1). In contrast, regardless of the inclusion of sucrose in the culture medium, fungal VCs did not stimulate growth of *cfbp1* plants (Figure 1). Fungal VCs did not promote growth in WT plants cultured in sucrose-containing medium under dark conditions (Supplementary Figure 1), strongly indicating that photosynthesis plays an important role in plant responses to fungal VCs.

### Fungal VCs Do Not Increase Photosynthetic Capacities of Exposed *cfbp1* Plants

**FIGURE 2:**
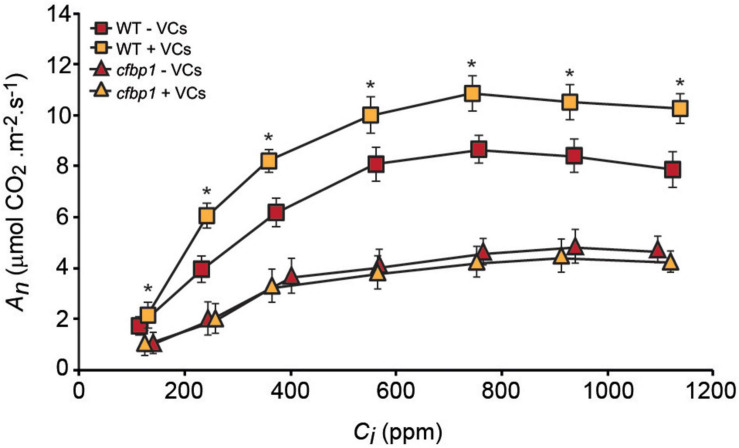
Fungal VCs do not increase photosynthetic capacities of exposed *cfbp1* plants. Curve of net CO_2_ assimilation rate (*A*_n_) vs. intercellular CO_2_ concentration (*C*_i_) in leaves of WT and *cfbp1* plants cultured in the absence or continuous presence of small VCs released by adjacent *A. alternata* cultures for 3 days. Treatment started 28 days after sowing plants. Values are means ±SE for four plants. Asterisks indicate significant differences relative to WT plants not cultured with small VCs released by adjacent fungal cultures according to Student’s *t*-test (*P* < 0.05).

To determine whether the non-perturbation of *cfbp1* plants by small fungal VCs could be due to the lack of a photosynthetic response to fungal VCs, key photosynthetic parameters were compared in fully developed WT and *cfbp1* plants cultured in the absence or continuous presence of adjacent fungal cultures. Under non-induced conditions, *A*_n_ at all *C*_i_ levels as well as *V*_cmax_, *J*_max_, and TPU values were all lower in *cfbp1* plants than in WT plants (Figure 2 and Table 1), which is consistent with Rojas-González et al. (2015). Leaves of fungal VC-treated WT plants had higher *A*_n_ values than controls at all *C*_i_ levels (Figure 2) as found by Sánchez-López et al. (2016b). Additionally, fungal VC-treated WT leaves had higher *V*_cmax_, TPU, and *J*_max_ values than non-treated controls (Table 1). In contrast, no significant differences were observed in *A*_n_ values between fungal VC-treated and non-treated *cfbp1* leaves at any *C*_i_ levels (Figure 2). Consistently, no significant differences in *V*_cmax_, TPU, and *J*_max_ values were observed between VC-treated and non-treated *cfbp1* leaves (Table 1).

**TABLE 1:** Photosynthetic parameters of leaves of WT and *cfbp1* plants cultured in the absence or continuous presence of VCs emitted by adjacent *A. alternata* cultures covered with charcoal filters for 3 days.

	*V*_cmax_ (μ mol e^–1^ m^–2^ s^–1^)	*J*_max_ (μ mol CO_2_ m^–2^ s^–1^)	*TPU* (μ mol Pi m^–2^ s^–1^)
WT - VCs	20.2 ± 0.4	50.2 ± 1.3	1.11 ± 0.17
WT + VCs	25.4 ± 0.6*	63.9 ± 1.7*	1.54 ± 0.05*
*cfbp1* - VCs	11.7 ± 0.6	28.5 ± 3.4	0.67 ± 0.13
*cfbp1* + VCs	12.3 ± 0.3	29.5 ± 2.5	0.68 ± 0.09

*Values are means ± SE for three biological replicates (each a pool of four plants) obtained from four independent experiments. Asterisks indicate significant differences relative to plants not cultured with adjacent fungal cultures according to Student’s t-test (P < 0.05).*

### Exposure to Fungal VCs Does Not Alter the Levels of Compounds Derived From the MEP and Shikimate Pathways in *cfbp1* Leaves

**FIGURE 3:**
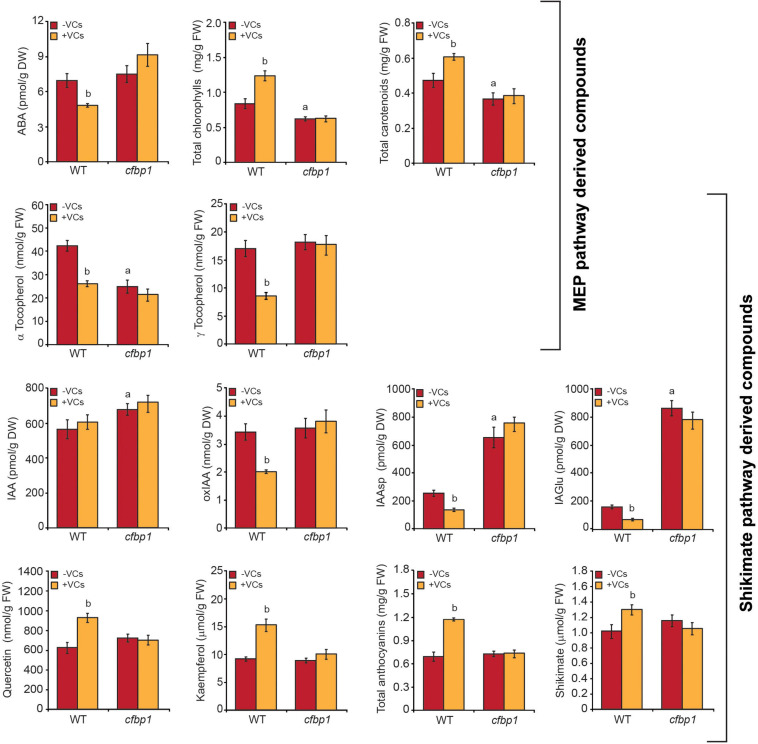
Fungal VCs do not alter the contents of MEP and shikimate pathway-derived compounds in leaves of *cfbp1* plants. The graphics represent the contents of MEP and shikimate pathway-derived compounds in leaves of WT and *cfbp1* plants cultured in agar solidified MS medium without sucrose supplementation in the absence or continuous presence of small VCs released by adjacent *A. alternata* cultures for 3 days. Values are means ±SE for three biological replicates (each comprising a pool of 12 plants) from three independent experiments. ^a^Significant differences, according to Student’s *t*-test (*P* < 0.05), between WT and *cfbp1* plants cultured without fungal VC treatment. ^b^Significant differences, according to Student’s *t*-test (*P* < 0.05), between VC-treated and non-treated WT plants.

We next compared the effects of small fungal VCs on contents of MEP pathway-derived compounds (e.g., ABA, CK, tocopherols, chlorophylls, and carotenoids) and shikimate pathway-derived compounds such as shikimate, phenylpropanoids (e.g., flavonols and anthocyanins), IAA, and IAA conjugates in mature leaves of WT and *cfbp1* plants. In the absence of fungal VCs, levels of CK, ABA, γ-tocopherol, flavonols (e.g., kaempherol and quercetin), and total anthocyanins in *cfbp1* leaves were similar to those in WT leaves, while levels of IAA and IAA conjugates were higher than in WT leaves and levels of α-tocopherol were lower (Figure 3, Supplementary Figure 2, and Supplementary Table 2). In keeping with Rojas-González et al. (2015), *cfbp1* leaves accumulated lower levels of total chlorophylls and carotenoids than WT leaves (Figure 3). As shown in Figure 3 and Supplementary Figure 2, *A. alternata* VCs caused a significant increase in contents of chlorophylls, carotenoids, shikimate, anthocyanins, and flavonols, and a reduction in contents of ABA, tocopherols, and IAA conjugates in WT leaves, but had no effect in *cfbp1* leaves. Moreover, fungal VCs enhanced the contents of precursors, active, and transport forms of plastidial CKs in leaves of both WT and *cfbp1* plants (Supplementary Table 2).

### Fungal VCs Exposure Weakly Alter the Proteome of *cfbp1* Leaves

We performed high-throughput differential proteomic analysis of leaves of WT and *cfbp1* Arabidopsis plants cultured in the absence or presence for 3 days of VCs emitted by adjacent *A. alternata* cultures. Because some responses promoted by fungal VCs could be due to fine regulation of the proteome, this analysis was conducted using a label-free strategy based on spectral counting and ion intensity, which is known to provide deeper proteome coverage for identifying differentially expressed proteins (DEPs) among available quantitative proteomic methods (Megger et al., 2014).

As shown in Supplementary Tables 3, 4, 1,054 and 68 out of the 3,135 proteins identified in the study of WT plants (Supplementary Table 5) were proteins with known functions that were differentially expressed upon exposure to fungal VCs with statistical significance levels of “confident” (established at *q* value levels ≤0.01) and “likely” (established at *q* value levels >0.01 and ≤0.05), respectively. Among the 1,054 proteins for which differential expression following VC exposure was established at the “confident” significance level, 569 were up-regulated and 485 were down-regulated (Supplementary Table 3). Unlike in WT plants, fungal VCs did not cause changes in the expression of proteins at the “confident” statistical significance level in *cfbp1* plants, and only 298 out of the 3,168 proteins identified in the study plants were differentially expressed with a “likely” statistical significance level (Supplementary Tables 6, 7). Proteomic analysis of leaves of WT and *cfbp1* plants not exposed to fungal VCs revealed that knocking out cFBP1 causes important changes in the leaf proteome (Supplementary Figure 3 and Supplementary Tables 8, 9). Notably, it disturbs the expression of proteins involved in the plastidial PQC system and down-regulates that of plastidial thioredoxins and enzymes related to the MEP pathway and photosynthesis (Supplementary Figure 3 and Supplementary Tables 8, 9).

### Fungal VCs Alter the Expression of MEP and Shikimate Pathway Enzymes and of Proteins Involved in the Plastid Proteostasis in WT Plants

**FIGURE 4:**
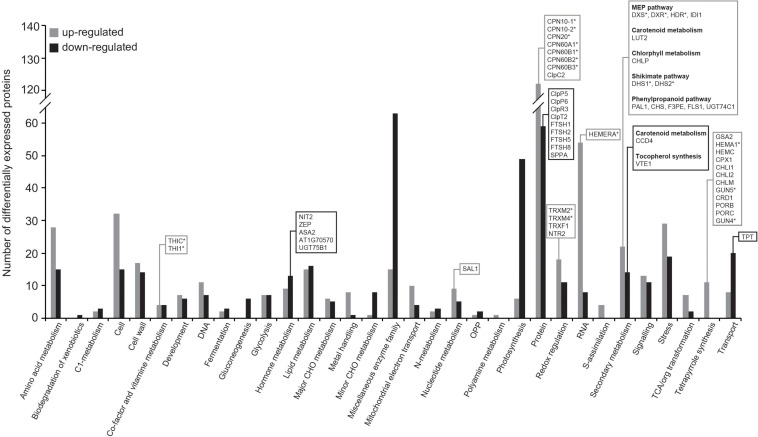
Fungal VCs alter the expression of enzymes of the MEP and shikimate pathways and of proteins involved in the plastidial proteostasis in WT plants. The graphic represents the functional categorization of differentially expressed proteins (DEPs) in leaves of WT plants cultured in the presence of small VCs emitted by adjacent *A. alternata* cultures for 3 days. The proteins that were significantly down- and up-regulated following VC exposure were arranged according to the putative functional category assigned by MapMan software. The numbers of up- and down-regulated proteins in each categorical group are indicated by gray and black bars, respectively. Proteins discussed here are boxed, and putative Clp clients are indicated with asterisks. Data obtained from Supplementary Table 3.

Using the broad characterizations outlined by the MapMan tool^1^ (Thimm et al., 2004), the 1,054 proteins differentially expressed upon exposure to fungal VCs in WT leaves were assembled into 33 functional groups (Figure 4). Predicted locations of these proteins obtained using the SUBA4 Arabidopsis protein subcellular localization database (Hooper et al., 2017) included almost all cellular compartments, but the location associated with the greatest number of proteins was the plastids (Supplementary Table 3 and Supplementary Figure 4). Fungal VCs enhanced the expression of plastidial proteins including redox enzymes (e.g., TRXM2, TRXM4, TRXF1, and NTR2), MEP pathway enzymes (e.g., DXS, DXR, HDR, and IDI1), enzymes involved in the synthesis of chlorophyll (e.g., GSA2, HEMA1, HEMC, CPX1, CHLI1, CHLI2, CHLM, GUN5, CRD1, PORB, PORC, GUN4, and CHLP) and carotenoids (e.g., LUT2), shikimate pathway enzymes (e.g., DHS1 and DHS2), and phenylpropanoid pathway enzymes (e.g., PAL1, CHS, F3PE, FLS1, and UGT74C1) (Figure 4 and Supplementary Table 3). Fungal VCs also down-regulated the expression of enzymes involved in carotenoid breakdown (e.g., CCD4), the synthesis of ABA from carotenoids (e.g., ZEP), and the conversion of chorismate into tocopherols (e.g., VTE1) and IAA and IAA conjugates (e.g., ASA2, At1g70570, NIT2, and UGT75B1) (Figure 4 and Supplementary Table 3). Except for CCD4, ZEP, F3PE, PAL1, FLS1, and CHS, none of these proteins are encoded by fungal VC-responsive genes (cf. Supplementary Table 3 in Sánchez-López et al. (2016b), indicating that changes in the leaf proteome promoted by fungal VCs are subject to non-transcriptional regulation. No changes in the expression of enzymes directly involved in CK metabolism were observed upon fungal VC treatment.

Fungal VC exposure also altered the expression of proteins of the plastidial PQC system. Specifically, fungal VCs reduced the expression of four plastidial ATP-dependent zinc metalloproteases (e.g., FTSH1, FTSH2, FTSH5, and FTSH8), one serine protease (e.g., SPPA), several subunits of the proteolytic core of the ATP-dependent serine-type Clp protease complex (e.g., ClpP5, ClpP6, and ClpR3), and the Clp stabilizer ClpT2 (Figure 4 and Supplementary Table 3). VC exposure also increased the expression of chaperones that are implicated in the folding and onward guidance of newly imported plastid proteins into their native states, including four of the six plastidial chaperonins (e.g., CPN60A1, CPN60B1, CPN60B2, and CPN60B3) and the three plastidial co-chaperonins of Arabidopsis (e.g., CPN20, CPN10-1, and CPN10-2) (Kessler and Blobel, 1996; Tsai et al., 2012; Figure 4 and Supplementary Table 3).

### Fungal VCs Alter the Expression of Genes Encoding Proteins of the PQC System in WT Plants

**FIGURE 5:**
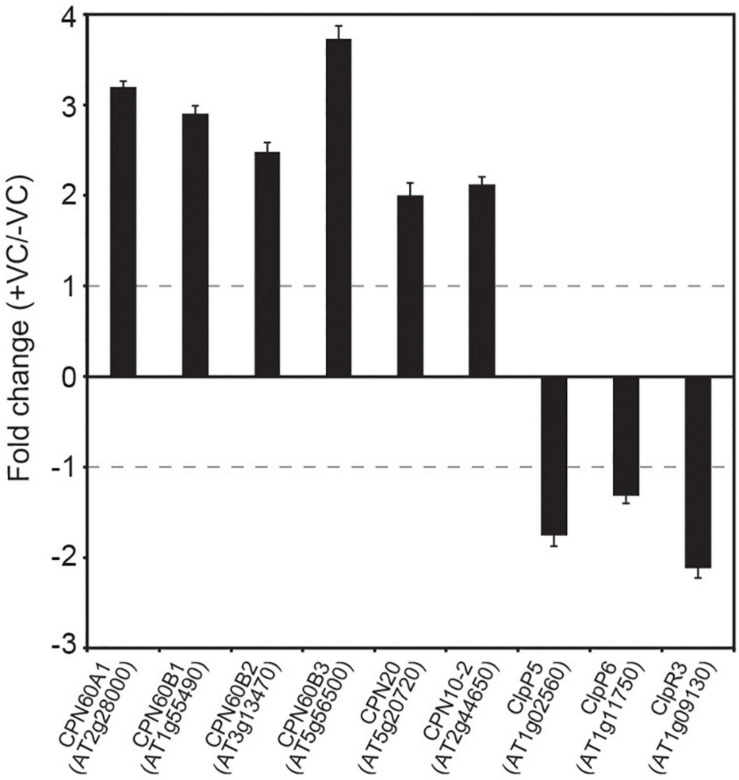
Fungal VCs alter the expression of genes encoding proteins of the PQC system in WT plants. Relative abundance of transcripts encoding PQC system proteins differentially expressed following exposure to small fungal VCs in WT leaves. Fold change values are differences in levels of transcripts (measured by quantitative RT-PCR) in leaves of WT plants cultured in the presence of small fungal VCs for 3 days relative to those in leaves of plants cultured in the absence of VCs. Gray dashed lines indicate the threshold of log2-fold change = 1 (twofold change) used to identify genes significantly regulated by fungal VCs. Values are means ± SE for three biological replicates.

Regulation of the MEP pathway requires chloroplast-to-nucleus communication that involves Clp protease-regulated expression of nuclear genes encoding plastid-targeted chaperones (Rodríguez-Concepción et al., 2019). Changes in the levels of Clp protease components and plastidial chaperonins and co-chaperonins promoted by small fungal VCs in WT plants but not in *cfbp1* plants, suggested chloroplast-to-nucleus signaling of enhanced photosynthesis. To test this hypothesis, we used qRT-PCR to analyze levels of transcripts encoding fungal VC-responsive proteins of the PQC system in WT and *cfbp1* plants cultured in the absence or presence of fungal VCs. These analyses revealed that small fungal VCs up-regulated the expression of *CPN60A1, CPN60B1, CPN60B2, CPN60B3, CPN20*, and *CPN10-2*, and down-regulated the expression of *ClpP4, ClpP5*, and *ClpR3* in WT plants (Figure 5). As shown in Supplementary Figure 5, fungal VCs did not enhance the expression of genes encoding plastidial chaperonins and co-chaperonins in *cfbp1* plants.

### Plants With Disrupted Plastidial Protein Homeostasis Respond Weakly to Small Fungal VCs

**FIGURE 6:**
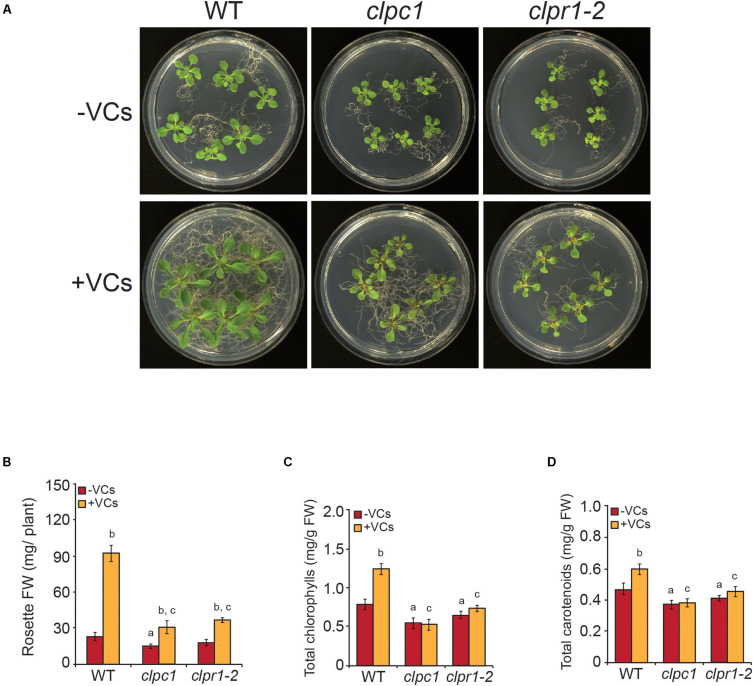
Plants with disrupted plastidial protein homeostasis weakly respond to fungal VCs. **(A)** External phenotypes and **(B)** rosette FW of WT, *clpc1*, and *clpr1-2* plants cultured in the absence or continuous presence of small fungal VCs for 1 week. Total contents of **(C)** chlorophylls and **(D)** carotenoids in leaves of WT, *clpc1*, and *clpr1-2* plants cultured in the absence or continuous presence of adjacent *A. alternata* cultures for 3 days. Values in “B” and “C” and “D” are means ± SE for three biological replicates (each comprising a pool of 12 plants) from four independent experiments. Letters “a,” “b,” and “c” indicate significant differences, according to Student’s *t*-test (*P* < 0.05), between (a) WT plants and mutants cultured without fungal VC treatment, (b) VC-treated and non-treated plants, and (c) VC-treated WT and mutant plants.

Plants with decreased Clp proteolytic capacity accumulate high levels of DXS, DXR, HDR, DHS1, DHS2, THIC, THI1, HEMERA, HEMA1, GUN4, GUN5, TRXM2, TRXM4, ClpC2, CPN60A1, CPN60B1, CPN60B2, CPN60B3, CPN20, CPN10-1, and CPN10-2 (Rudella et al., 2006; Zybailov et al., 2009; Nishimura et al., 2013; Pulido et al., 2016; Moreno et al., 2018; Rodríguez-Concepción et al., 2019). Up-regulation of these proteins and down-regulation of ClpP5, ClpP6, ClpR3, and ClpT2 promoted by small fungal VCs (Figure 4 and Supplementary Table 3) suggested that fine regulation of plastid proteostasis plays important roles in plant responses to microbial VCs. To test this hypothesis, we characterized the responses of *clpr1-2* and *clpc1* mutants defective in Clp protease activity and in the Hsp100 chaperone ClpC1 that delivers protein clients to the Clp catalytic core, respectively, to small fungal VCs. In the absence of fungal VCs, both *clpr1-2* and *clpc1* plants were slightly smaller and accumulated less photosynthetic pigments than WT plants (Figure 6), as reported by Welsch et al. (2018). Notably, fungal VC-promoted growth was substantially weaker in the two mutants than in WT plants (Figure 6). Furthermore, unlike in WT plants, VCs did not enhance total chlorophylls and carotenoids in the mutants (Figure 6).

## Discussion

### Redox Activation of Photosynthetic CO_2_ Fixation Is an Important Determinant of Plant Responses to Small Microbial VCs

We recently showed that small microbial VCs promote global reduction of the thiol redox proteome in WT leaves (with particularly strong effects on CBC enzymes), which may partly explain the stimulation of growth and photosynthesis in plants by VCs (Ameztoy et al., 2019). Here, we found that, unlike in WT and redox-sensitive cFBP1-expressing *cfbp2* plants, fungal VCs did not stimulate growth and photosynthesis in redox-insensitive cFBP2-expressing *cfbp1* plants. Furthermore, fungal VCs did not alter the levels of some shikimate and MEP pathway-derived compounds in *cfbp1* leaves. Moreover, unlike in WT plants, fungal VCs weakly promoted changes in the proteome of *cfbp1* leaves. Finally, fungal VCs did not promote growth of WT plants cultured in sucrose-containing medium under continuous dark conditions. These observations indicate that enhancement of photosynthetic CO_2_ fixation through light-dependent redox activation of CBC enzymes is an important determinant of the plants’ growth, proteomic, and metabolic responses to small fungal VCs.

### Proteome Resetting Can Account for Enhanced Photosynthetic CO_2_ Fixation and Changes in the Contents of MEP and Shikimate Pathway-Derived Compounds Promoted by Small Fungal VCs

**FIGURE 7:**
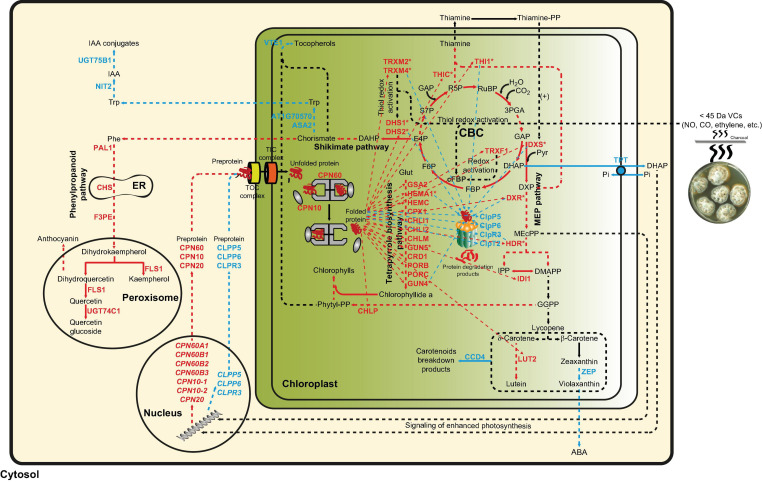
Suggested model of regulation of the plant response to small fungal VCs involving proteostatic modulation of MEP and shikimate pathways by redox-activated photosynthesis signaling. According to this model, the response of plants to small fungal VCs involves post-translational regulatory mechanisms wherein signaling of VC-promoted redox activation of photosynthesis and subsequent PQC system-mediated proteostatic up-regulation of enzymes of the MEP and shikimate pathways play important roles. Thiol redox activation of photosynthesis-related proteins promoted by microbial VCs increases the production of GAP and E4P, which enter the MEP and shikimate pathways, respectively, to fuel the production of compounds that initiate a cascade of signaling reactions leading to changes in the expression of nuclear encoded plastidial PQC system functions. Reduced protease (e.g., FTSH and Clp) activity and enhanced expression of chaperones in the chloroplast increase the stability and activity of enzymes of the MEP, shikimate, and tetrapyrrole biosynthesis pathways, creating a high metabolic flux toward the production of secondary metabolites important for growth and development including hormones, photosynthetic pigments, and ROS scavengers. The high chaperone-to-protease balance also promotes the accumulation of active thioredoxins that redox activates enzymes of the CBC, shikimate, and tetrapyrrole biosynthesis pathways. Enzymatic activities up-regulated by small fungal VCs are highlighted with red letters, while enzymatic activities and pathways down-regulated by fungal VCs are highlighted with blue letters. Multistep enzymatic reactions and signaling cascades are indicated by dashed arrows.

Data presented in this work indicate that fungal VC-promoted enhancement of photosynthetic CO_2_ fixation and changes in the levels of MEP and shikimate pathway-derived compounds in WT plants are at least partly due to proteome and redox-proteome resetting, as shown schematically in Figure 7. Enhanced photosynthetic CO_2_ fixation promoted by small fungal VCs could potentially be due to redox activation of the CBC caused by up-regulation of TRXM2, TRXM4, and TRXF1 because these thioredoxins are major determinants of the light-dependent redox activation of the CBC enzymes (Okegawa and Motohashi, 2015; Naranjo et al., 2016). Fungal VC-promoted photosynthesis can also be ascribed to enhanced production of chlorophylls and carotenoids caused by (i) down-regulation of carotenoid degradation enzymes; (ii) up-regulation of MEP pathway enzymes and enzymes involved in the synthesis of tetrapyrroles, phytyl-PP, and carotenoids; and (iii) up-regulation of TRXM2 and TRXM4, as these thioredoxins activate enzymes of the tetrapyrrole biosynthetic pathway (Da et al., 2017). Enhanced contents of shikimate, flavonols, and anthocyanins promoted by fungal VCs could at least partly be due to activation of the shikimate pathway caused by (i) increased expression of the thioredoxins TRXM2 and TRXM4, which redox-activate this pathway’s main regulatory enzymes (e.g., DHS1 and DHS2) (Entus et al., 2002); and (ii) up-regulation of the expression of DHS1 and DHS2 and phenylpropanoid pathway enzymes (e.g., PAL1, CHS, F3PE, and FLS1). In addition, fungal VC-promoted reduction of the levels of tocopherols and IAA conjugates can be ascribed to down-regulation of the expression of enzymes involved in converting chorismate into tocopherol (e.g., VTE1), and IAA and its conjugates (e.g., ASA2, At1g70570, NIT2, and UGT75B1). The reduction of ABA levels promoted by fungal VCs (Figure 3) is consistent with down-regulation of ZEP expression. Enhanced contents of plastidial CKs in VC-exposed WT and *cfbp1* plants suggests that VC-promoted enhancement of CK content is not due to enhanced flux from the CBC to the MEP pathway or to changes in the expression of CK biosynthetic enzymes in leaves (discussed in Supplementary Appendix).

### The Plastid Protein Quality Control System Mediates Changes in the Chloroplast Proteome in Response to Small Fungal VCs

Proteases (including Clp, FtsH, Lon, and Deg) and chaperones (including HSP70/DnaJ, HSP90, Hsp100, and CPN60/CPN10) are essential components of the PQC system in chloroplasts and are important for fine regulation of photosynthesis, metabolism, and signaling pathways as well as viability of cells and chloroplasts (Nishimura et al., 2017; Zhao and Liu, 2018; Rodríguez-Concepción et al., 2019). Enzymes of the MEP and shikimate pathways; enzymes involved in the synthesis of tetrapyrroles, carotenoids, and thiamine; and plastidial proteins involved in phytochrome signaling and redox regulation are putative clients of the Clp protease system (Zybailov et al., 2009; Nishimura et al., 2013; Pulido et al., 2016; Rodríguez-Concepción et al., 2019). Using highly sensitive label-free high-throughput differential proteomic analysis, here we showed that small fungal VCs reduce the expression of proteins of the Clp protease system (e.g., ClpP5, ClpP6, ClpR3, and ClpT2). This was surprising as these proteins are constitutively expressed and show only minor changes in expression under long-term stress conditions (Zheng et al., 2002). In addition, we found that plastidial proteins that accumulate to higher levels in Clp-defective mutants than in WT plants also accumulate to higher levels in fungal VC-treated plants than in controls. Proteins in this group include enzymes involved in the MEP pathway (e.g., DXS, DXR, and HDR), the shikimate pathway (e.g., DHS1 and DHS2), thiamine–PP synthesis (e.g., THIC and THI1), phytochrome signaling (e.g., HEMERA), tetrapyrrole synthesis (HEMA1, GUN4, and GUN5), redox regulation (e.g., TRXM2 and TRXM4), and folding and assembly of newly imported plastid proteins (e.g., ClpC2, CPN60A1, CPN60B1, CPN60B2, CPN60B3, CPN20, CPN10-1, and CPN10-2) (Rudella et al., 2006; Zybailov et al., 2009; Nishimura et al., 2013; Pulido et al., 2016; Rodríguez-Concepción et al., 2019). This indicates that the adaptation of the chloroplast proteome to small fungal VCs is mediated by mechanisms regulating the plastidial PQC system wherein dynamic balances between the expression of chaperones, the Clp protease complex, and other proteases play important roles. The weak response to fungal VCs in *clpc1* and *clpr1-2* plants with disrupted plastidial protein homeostasis strongly indicates that plant responses to fungal VCs require a finely regulated plastidial PQC system.

### Plant Responses to Small Fungal VCs Involve Chloroplast-to-Nucleus Retrograde Signaling

Chloroplast-to-nucleus retrograde signaling is important for informing the nuclear genome about the physiological and functional state of the chloroplast under varying environmental cues and for adjusting the chloroplast proteome accordingly via mechanisms involving the PQC system (Chan et al., 2016; Hernández-Verdeja and Strand, 2018). Accumulation of nuclear gene-encoded stromal chaperones in mutants with reduced Clp proteolytic activity is a response that involves chloroplast retrograde signaling, as protein folding stress in the chloroplasts of plants with decreased Clp activity sends as-yet unidentified signals to the nucleus that up-regulate the expression of genes encoding plastidial chaperones (Llamas et al., 2017; Perlaza et al., 2019). In WT leaves, reduction of the expression of proteins of the Clp protease system (e.g., ClpP5, ClpP6, ClpR3, and ClpT2) promoted by small fungal VCs was associated with down-regulation of *ClpP5, ClpP6*, and *ClpR3* transcript levels and enhanced levels of CPN60A1, CPN60B1, CPN60B2, CPN60B3, CPN20, CPN10-1, and CPN10-2 and their encoding transcripts. No such increases in the expression of plastidial chaperonins and co-chaperonins occurred in leaves of *cfbp1* plants or in roots of WT plants exposed to small fungal VCs (García-Gómez et al., 2020). This indicates that, as illustrated in Figure 7, plastid proteome adaptation to fungal VCs in leaves involves photosynthesis-driven chloroplast-to-nucleus retrograde signaling mechanisms wherein transcriptional up-regulation of plastidial chaperones and down-regulation of the Clp protease system play important roles. To our knowledge, this is the first example of an environmentally triggered modulation of the expression of genes encoding Clp protease subunits as a way to change the levels of MEP and shikimate pathway enzymes and hence regulate chloroplast metabolism. The hypothesis that photosynthetic activity triggers retrograde signals in VC-exposed plants is supported by the fact that, contrary to the effect of fungal VCs on the plastidial proteome of WT plants, knocking out cFBP1 reduces photosynthetic activity and down-regulates the expression of plastidial chaperonins, co-chaperonins, MEP pathway enzymes, and thioredoxins in leaves.

Fungal VCs did not alter the expression of chaperones involved in DXS turnover (e.g., ClpC1, Hsp70, and ClpB3). Therefore, chloroplast-to-nucleus retrograde signaling pathways operating in VC-exposed plants appear to differ from those reported to control the expression of the model protein DXS in plants with reduced Clp activity (Pulido et al., 2013; Llamas et al., 2017).

Signals involved in chloroplast-to-nucleus retrograde signaling include reactive oxygen species (ROS) generated by photosynthesis, tetrapyrroles, the sulfur metabolism by-product 3′-phosphoadenosine 5′-phosphate (PAP), the MEP pathway intermediate 2-C-methyl-D-erythritol-2,4-cyclopyrophosphate, isoprenes, and the CBC intermediate dehydroxyacetone phosphate (Estavillo et al., 2011; Xiao et al., 2012; Vogel et al., 2014; Chan et al., 2016). Small fungal VCs do not promote changes in ROS levels in leaves (Ameztoy et al., 2019), suggesting that these compounds are not involved in retrograde signaling of redox-activated photosynthesis promoted by fungal VCs. Dehydroxyacetone phosphate produced by the CBC can be exported to the cytosol through the triose-phosphate/phosphate translocator (TPT), where it can activate protein phosphorylation signaling cascades (Estavillo et al., 2011). Fungal VCs down-regulated TPT expression, indicating that retrograde signaling of redox-activated photosynthesis promoted by fungal VCs does not involve enhanced TPT-mediated transport of dehydroxyacetone phosphate to the cytosol. In contrast, fungal VCs up-regulated the expression of MEP pathway enzymes and SAL1, a plastidial enzyme that controls the intracellular levels of PAP (Estavillo et al., 2011; Phua et al., 2018). Therefore, it is likely that chloroplast-to-nucleus retrograde signaling of enhanced photosynthesis promoted by small fungal VCs involves PAP and/or MEP pathway intermediates such as 2-C-methyl-D-erythritol-2,4-cyclopyrophosphate. An alternative or additional candidate actor in this process is HEMERA, the expression of which is up-regulated by fungal VCs. This transcription factor shows dual localization in the chloroplast nucleoids and the nucleus (Chen et al., 2010; Melonek et al., 2016), and is abundant in plants with reduced Clp activity, suggesting that its accumulation in chloroplasts may cause its translocation to the nucleus to regulate nuclear gene expression (Moreno et al., 2018). HEMERA participates in phytochrome signaling (Chen et al., 2010), and the accumulation of exceptionally high levels of starch promoted by fungal VCs is subject to phytochrome signaling (Li et al., 2011). The translocation of HEMERA may thus be involved in the chloroplast-to-nucleus retrograde signaling of fungal VCs.

### An Integrative Model of the Regulation of the Plant Response to Small Microbial VCs Involving Proteostatic Modulation of MEP and Shikimate Pathways by Redox-Activated Photosynthesis Signaling

Results presented in this and previous works (Ameztoy et al., 2019; García-Gómez et al., 2019) provide evidence that the response of plants to small fungal VCs involves transcriptional and post-translational regulatory mechanisms that depend heavily on signaling of redox-activated photosynthesis and subsequent PQC system-mediated proteostatic up-regulation of thioredoxins and enzymes of the MEP, shikimate, and tetrapyrrole biosynthesis pathways. According to this view (see Figure 7), rapid thiol redox activation of CBC enzymes promoted by microbial VCs augments the photosynthetic production of GAP and E4P. These compounds then enter the MEP and shikimate pathways, respectively, fueling the production of compounds that initiate a cascade of signaling reactions leading to changes in the expression of nuclear encoded plastidial PQC system functions. Reduced protease activity and enhanced expression of chaperones in the chloroplasts increase the stability and activity of enzymes of the MEP, shikimate, and tetrapyrrole biosynthesis pathways, creating a high metabolic flux from the CBC toward the production of secondary metabolites important for growth and development including hormones, photosynthetic pigments, ROS scavengers, etc. The high chaperone-to-protease balance also promotes the accumulation of thioredoxins, which in turn redox-activate enzymes of the CBC, shikimate, and tetrapyrrole biosynthesis pathways (Figure 7).

## Data Availability Statement

The original contributions presented in the study are included in the article/Supplementary Material, further inquiries can be directed to the corresponding author/s.

## Author Contributions

KA, MR-C, and JP-R designed the experiments, analyzed the data, and wrote the article with contributions from all the authors. ÁS-L, FJM, AB, GA, EB-F, SG-A, NDD, ON, AP, AA, and KD performed most of the experiments. JP-R supervised the experiments. JP-R conceived the project and research plans. All authors contributed to the article and approved the submitted version.

## Conflict of Interest

The authors declare that the research was conducted in the absence of any commercial or financial relationships that could be construed as a potential conflict of interest.

## Footnotes

1 https://mapman.gabipd.org/

## Supplementary Material

Supplementary Figure 1 External phenotypes and rosette FW of WT plants cultured in darkness in agar solidified MS medium supplemented with sucrose in the absence or continuous presence of small VCs released by adjacent *A. alternata* cultures for 1 week.

Supplementary Figure 2 Contents of MEP and shikimate pathways-derived compounds in leaves of WT and *cfbp1* plants cultured in agar solidified MS medium supplemented with sucrose in the absence or continuous presence of small VCs released by adjacent *A. alternata* cultures for 3 days.

Supplementary Figure 3 Functional categorization of the proteins that are differentially expressed upon knocking out cFBP1.

Supplementary Figure 4 Categorization of fungal VC-responsive proteins according to their subcellular localizations.

Supplementary Figure 5 Relative abundance of transcripts encoding plastidial chaperones differentially expressed by fungal VCs in *cfbp1* leaves.

Supplementary Table 1 Primers used in qRT-PCR.

Supplementary Table 2 CK content (pmol g^–1^ DW) in leaves of WT and *cfbp1* plants cultured in agar solidified MS medium in the absence or presence of small VCs emitted by *A. alternata* for 3 days.

Supplementary Table 3 List of proteins that are differentially expressed by small fungal VCs in leaves of WT plants with a “confident” statistical significance level.

Supplementary Table 4 List of proteins that are differentially expressed by small fungal VCs in leaves of WT plants with a “likely” statistical significance level.

Supplementary Table 5 List of proteins identified in the proteomic study in rosettes of WT plants cultured in the absence or presence of small fungal VCs.

Supplementary Table 6 List of proteins that are differentially expressed by fungal VCs in leaves of *cfbp1* plants with a “likely” statistical significance level (fungal VC-treated vs. non-treated plants). DEPs are classified by fold-change.

Supplementary Table 7 List of proteins identified in the differential proteomic analysis of *cfbp1* plants cultured in the absence or presence for 3 days of small VCs emitted by cultures of *Alternaria alternata.*

Supplementary Table 8 List of proteins that are differentially expressed in leaves by knocking down cFBP1 with a “confident” statistical significance level.

Supplementary Table 9 List of proteins identified in the differential proteomic analysis of WT and *cfbp1* plants cultured in the absence of small fungal VCs.

Supplementary Discussion The increase in cytokinin levels promoted by small fungal VCs is not due to an enhanced flux from the CBC to the MEP pathway.

## Abbreviations

ABA: abscisic acid
*A*_n_: net rate of CO_2_ assimilation
cFBP: fructose-1,6-bisphosphatase
*C*_i_: intracellular CO_2_ concentration
CK: cytokinin
DEPs: differentially expressed proteins
DHS: phospho-2-dehydro-3-deoxyheptonate aldolase
DXS: 1-deoxy-D-xylulose-5-phosphate synthase
GAP: glyceraldehyde 3-phosphate
IAA: indole acetic acid
*J*_max_: maximum electron transport demand for RuBP regeneration
PQC: protein quality control
MEP: 2-C-methyl-D-erythritol 4-phosphate
MS: Murashige and Skoog
PAP: 3 ′ -phosphoadenosine 5 ′ -phosphate
ROS: reactive oxygen species
TPT: triose-phosphate/phosphate translocator
TPU: the triose phosphate use
*V*_cmax_: maximum rate of carboxylation by Rubisco
VCs: volatile compounds
VOC: volatile organic compounds
WT: wild-type.

## References

[B1] Ameztoy K. Baslam M. Sánchez-López ÁM. Muñoz F. J. Bahaji A. Almagro G. (2019). Plant responses to fungal volatiles involve global post-translational thiol redox proteome changes that affect photosynthesis. *Plant. Cell Environ.* 42 2627–2644. 10.1111/pce.13601 31222760

[B2] Banerjee A. Wu Y. Banerjee R. Li Y. Yan H. Sharkey T. D. (2013). Feedback inhibition of deoxy-D-xylulose-5-phosphate synthase regulates the methylerythritol 4-phosphate pathway. *J. Biol. Chem.* 288 16926–16936. 10.1074/jbc.M113.464636 23612965 PMC3675625

[B3] Carretero-Paulet L. Ahumada I. Cunillera N. Rodríguez-Concepción M. Ferrer A. Boronat A. (2002). Expression and molecular analysis of the *Arabidopsis DXR* gene encoding 1-deoxy-D-xylulose 5-phosphate reductoisomerase, the first committed enzyme of the 2-C-methyl-D-erythritol 4-phosphate pathway. *Plant Physiol.* 129 1581–1591. 10.1104/pp.003798 12177470 PMC166745

[B4] Chan K. X. Phua S. Y. Crisp P. McQuinn R. Pogson B. J. (2016). Learning the languages of the chloroplast: retrograde signaling and beyond. *Annu. Rev. Plant Biol.* 67 25–53. 10.1146/annurev-arplant-043015-111854 26735063

[B5] Chen M. Galvão R. M. Li M. Burger B. Bugea J. Bolado J. (2010). *Arabidopsis* HEMERA/pTAC12 initiates photomorphogenesis by phytochromes. *Cell* 141 1230–1240. 10.1016/j.cell.2010.05.007 20603003 PMC2935685

[B6] Córdoba E. Salmi M. León P. (2009). Unravelling the regulatory mechanisms that modulate the MEP pathway in higher plants. *J. Exp. Bot.* 60 2933–2943. 10.1093/jxb/erp190 19584121

[B7] Da Q. Wang P. Wang M. Sun T. Jin H. Liu B. (2017). Thioredoxin and NADPH-dependent thioredoxin reductase c regulation of tetrapyrrole biosynthesis. *Plant Physiol.* 175 652–666. 10.1104/pp.16.01500 28827456 PMC5619880

[B8] Ditengou F. A. Müller A. Rosenkranz M. Felten J. Lasok H. Van Doorn M. M. (2015). Volatile signalling by sesquiterpenes from ectomycorrhizal fungi reprogrammes root architecture. *Nat. Commun.* 6:6279. 10.1038/ncomms7279 25703994 PMC4346619

[B9] Entus R. Poling M. Herrmann K. M. (2002). Redox regulation of *Arabidopsis* 3-deoxy-D-arabino-heptulosonate 7-phosphate synthase. *Plant Physiol.* 129 1866–1871. 10.1104/pp.002626 12177500 PMC166775

[B10] Estavillo G. M. Crisp P. A. Pornsiriwong W. Wirtz M. Collinge D. Carrie C. (2011). Evidence for a SAL1-PAP chloroplast retrograde pathway that functions in drought and high light signaling in *Arabidopsis*. *Plant Cell* 23 3992–4012. 10.1105/tpc.111.091033 22128124 PMC3246320

[B11] Floková K. Tarkowská D. Miersch O. Strnad M. Wasternack C. Novák O. (2014). UHPLC-MS/MS based target profiling of stress-induced phytohormones. *Phytochemistry* 105 147–157. 10.1016/j.phytochem.2014.05.015 24947339

[B12] García-Gómez P. Almagro G. Sánchez-López ÁM. Bahaji A. Ameztoy K. Ricarte-Bermejo A. (2019). Volatile compounds other than CO_2_ emitted by different microorganisms promote distinct posttranscriptionally regulated responses in plants. *Plant Cell Environ.* 42 1729–1746. 10.1111/pce.13490 30480826

[B13] García-Gómez P. Bahaji A. Gámez-Arcas S. Muñoz F. J. Sánchez-lópez ÁM. Almagro G. (2020). Volatiles from the fungal phytopathogen *Penicillium aurantiogriseum* modulate root metabolism and architecture through proteome resetting. *Plant Cell Environ.* 43 2551–2570. 10.1111/pce.13817 32515071

[B14] Garnica-Vergara A. Barrera-Ortiz S. Muñoz-Parra E. Raya-González J. Méndez-Bravo A. Macías-Rodríguez L. (2016). The volatile 6-pentyl-2H-pyran-2-one from *Trichoderma atroviride* regulates *Arabidopsis thaliana* root morphogenesis via auxin signaling and ETHYLENE INSENSITIVE 2 functioning. *New Phytol.* 209 1496–1512. 10.1111/nph.13725 26568541

[B15] Ghirardo A. Wright L. P. Bi Z. Rosenkranz M. Pulido P. Rodríguez-Concepción M. (2014). Metabolic flux analysis of plastidic isoprenoid biosynthesis in poplar leaves emitting and nonemitting isoprene. *Plant Physiol.* 165 37–51. 10.1104/pp.114.236018 24590857 PMC4012595

[B16] Ghirardo A. Zimmer I. Brüggemann N. Schnitzler J. P. (2010). Analysis of 1-deoxy-d-xylulose 5-phosphate synthase activity in Grey poplar leaves using isotope ratio mass spectrometry. *Phytochemistry* 71 918–922. 10.1016/j.phytochem.2010.02.016 20303132

[B17] Guo Y. Jud W. Ghirardo A. Antritter F. Benz J. P. Schnitzler J.-P. (2020). Sniffing fungi – phenotyping of volatile chemical diversity in *Trichoderma* species. *New Phytol.* 227 244–259. 10.1111/nph.16530 32155672

[B18] Henkes S. Sonnewald U. Badur R. Flachmann R. Stitt M. (2001). A small decrease of plastid transketolase activity in antisense tobacco transformants has dramatic effects on photosynthesis and phenylpropanoid metabolism. *Plant Cell* 13 535–552. 10.1105/tpc.13.3.535 11251095 PMC135503

[B19] Hernández-Verdeja T. Strand Å (2018). Retrograde signals navigate the path to chloroplast development. *Plant Physiol* 176 967–976. 10.1104/pp.17.01299 29254985 PMC5813530

[B20] Hooper C. M. Castleden I. R. Tanz S. K. Aryamanesh N. Millar A. H. (2017). SUBA4: The interactive data analysis centre for *Arabidopsis* subcellular protein locations. *Nucleic Acids Res.* 45 D1064–D1074. 10.1093/nar/gkw1041 27899614 PMC5210537

[B21] Kessler F. Blobel G. (1996). Interaction of the protein import and folding machineries in the chloroplast. *Proc. Natl. Acad. Sci. U. S. A.* 93 7684–7689. 10.1073/pnas.93.15.7684 8755536 PMC38807

[B22] Li J. Ezquer I. Bahaji A. Montero M. Ovecka M. Baroja-Fernández E. (2011). Microbial volatile-induced accumulation of exceptionally high levels of starch in *Arabidopsis* leaves is a process involving NTRC and starch synthase classes III and IV. *Mol. Plant Microbe Interact.* 24 1165–1178. 10.1094/MPMI-05-11-0112 21649509

[B23] Lichtenthaler H. K. (1987). Chlorophylls and carotenoids: pigments of photosynthetic biomembranes. *Methods Enzymol.* 148 350–382. 10.1016/0076-6879(87)48036-1

[B24] Llamas E. Pulido P. Rodríguez-Concepción M. (2017). Interference with plastome gene expression and Clp protease activity in *Arabidopsis* triggers a chloroplast unfolded protein response to restore protein homeostasis. *PLoS Genet.* 13:e1007022. 10.1371/journal.pgen.1007022 28937985 PMC5627961

[B25] Long S. P. Bernacchi C. J. (2003). Gas exchange measurements, what can they tell us about the underlying limitations to photosynthesis? Procedures and sources of error. *J. Exp. Bot.* 54 2393–2401. 10.1093/jxb/erg262 14512377

[B26] Megger D. A. Pott L. L. Ahrens M. Padden J. Bracht T. Kuhlmann K. (2014). Comparison of label-free and label-based strategies for proteome analysis of hepatoma cell lines. *Biochim. Biophys. Acta Proteins Proteom.* 1844 967–976. 10.1016/j.bbapap.2013.07.017 23954498

[B27] Melonek J. Oetke S. Krupinska K. (2016). Multifunctionality of plastid nucleoids as revealed by proteome analyses. *Biochim. Biophys. Acta Proteins Proteom.* 1864 12828–12831. 10.1016/j.bbapap.2016.03.009 26987276

[B28] Michelet L. Zaffagnini M. Morisse S. Sparla F. Pérez-Pérez M. E. Francia F. (2013). Redox regulation of the Calvin-Benson cycle: something old, something new. *Front. Plant Sci.* 4:479. 10.3389/fpls.2013.00470 24324475 PMC3838966

[B29] Moreno J. C. Martínez-Jaime S. Schwartzmann J. Karcher D. Tillich M. Graf A. (2018). Temporal proteomics of inducible RNAi lines of Clp protease subunits identifies putative protease substrates. *Plant Physiol.* 176 1485–1508. 10.1104/pp.17.01635 29229697 PMC5813558

[B30] Naranjo B. Diaz-Espejo A. Lindahl M. Cejudo F. J. (2016). Type-*f* thioredoxins have a role in the short-term activation of carbon metabolism and their loss affects growth under short-day conditions in *Arabidopsis thaliana*. *J. Exp. Bot.* 67 1951–1964. 10.1093/jxb/erw017 26842981 PMC4783373

[B31] Nishimura K. Asakura Y. Friso G. Kim J. Oh S. H. Rutschow H. (2013). ClpS1 is a conserved substrate selector for the chloroplast Clp protease system in *Arabidopsis*. *Plant Cell* 25 2276–2301. 10.1105/tpc.113.112557 23898032 PMC3723626

[B32] Nishimura K. Kato Y. Sakamoto W. (2017). Essentials of proteolytic machineries in chloroplasts. *Mol. Plant* 10 4–19. 10.1016/j.molp.2016.08.005 27585878

[B33] Novák O. Hauserová E. Amakorová P. Doležal K. Strnad M. (2008). Cytokinin profiling in plant tissues using ultra-performance liquid chromatography-electrospray tandem mass spectrometry. *Phytochemistry* 69 2214–2224. 10.1016/j.phytochem.2008.04.022 18561963

[B34] Okegawa Y. Motohashi K. (2015). Chloroplastic thioredoxin *m* functions as a major regulator of Calvin cycle enzymes during photosynthesis *in vivo*. *Plant J.* 84 900–913. 10.1111/tpj.13049 26468055

[B35] Pěnčík A. Rolčík J. Novák O. Magnus V. Barták P. Buchtík R. (2009). Isolation of novel indole-3-acetic acid conjugates by immunoaffinity extraction. *Talanta* 80 651–655. 10.1016/j.talanta.2009.07.043 19836533

[B36] Perlaza K. Toutkoushian H. Boone M. Lam M. Iwai M. Jonikas M. C. (2019). The Mars1 kinase confers photoprotection through signaling in the chloroplast unfolded protein response. *Elife* 8:e49577. 10.7554/eLife.49577 31612858 PMC6794094

[B37] Phua S. Y. Yan D. Chan K. X. Estavillo G. M. Nambara E. Pogson B. J. (2018). The Arabidopsis SAL1-PAP pathway: a case study for integrating chloroplast retrograde, light and hormonal signaling in modulating plant growth and development? *Front. Plant Sci.* 9:1171. 10.3389/fpls.2018.01171 30135700 PMC6092573

[B38] Pokhilko A. Bou-Torrent J. Pulido P. Rodríguez-Concepción M. Ebenhöh O. (2015). Mathematical modelling of the diurnal regulation of the MEP pathway in Arabidopsis. *New Phytol.* 206 1075–1085. 10.1111/nph.13258 25598499

[B39] Pulido P. Llamas E. Llorente B. Ventura S. Wright L. P. Rodríguez-Concepción M. (2016). Specific Hsp100 chaperones determine the fate of the first enzyme of the plastidial isoprenoid pathway for either refolding or degradation by the stromal Clp protease in *Arabidopsis*. *PLoS Genet.* 12:e1005824. 10.1371/journal.pgen.1005824 26815787 PMC4729485

[B40] Pulido P. Toledo-Ortiz G. Phillips M. A. Wright L. P. Rodríguez-Concepción M. (2013). Arabidopsis J-Protein J20 delivers the first enzyme of the plastidial isoprenoid pathway to protein quality control. *Plant Cell* 25 4183–4194. 10.1105/tpc.113.113001 24104567 PMC3877790

[B41] Rodríguez-Concepción M. D’Andrea L. Pulido P. (2019). Control of plastidial metabolism by the Clp protease complex. *J. Exp. Bot.* 70 2049–2058. 10.1093/jxb/ery441 30576524

[B42] Rojas-González J. A. Soto-Súarez M. García-Díaz Á Romero-Puertas M. C. Sandalio L. M. Mérida Á, et al. (2015). Disruption of both chloroplastic and cytosolic FBPase genes results in a dwarf phenotype and important starch and metabolite changes in *Arabidopsis thaliana*. *J. Exp. Bot.* 66 2673–2689. 10.1093/jxb/erv062 25743161 PMC4986871

[B43] Rudella A. Friso G. Alonso J. M. Ecker J. R. Van Wijk K. J. (2006). Downregulation of ClpR2 leads to reduced accumulation of the ClpPRS protease complex and defects in chloroplast biogenesis in *Arabidopsis*. *Plant Cell* 18 1704–1721. 10.1105/tpc.106.042861 16766689 PMC1488914

[B44] Ryu C.-M. Farag M. A. Hu C.-H. Reddy M. S. Wei H.-X. Pare P. W. (2003). Bacterial volatiles promote growth in *Arabidopsis*. *Proc. Natl. Acad. Sci.U.S.A.* 100 4927–4932. 10.1073/pnas.0730845100 12684534 PMC153657

[B45] Sánchez-López ÁM. Bahaji A. De Diego N. Baslam M. Li J. Muñoz F. J. (2016a). Arabidopsis responds to *Alternaria alternata* volatiles by triggering plastid phosphoglucose isomerase-independent mechanisms. *Plant Physiol.* 172 1989–2001. 10.1104/pp.16.00945 27663407 PMC5100789

[B46] Sánchez-López ÁM. Baslam M. De Diego N. Muñoz F. J. Bahaji A. Almagro G. (2016b). Volatile compounds emitted by diverse phytopathogenic microorganisms promote plant growth and flowering through cytokinin action. *Plant Cell Environ.* 39 2592–2608. 10.1111/pce.12759 27092473

[B47] Serrato A. J. Yubero-Serrano E. M. Sandalio L. M. Muñoz-Blanco J. Chueca A. Caballero J. L. (2009). cpFBPaseII, a novel redox-independent chloroplastic isoform of fructose-1,6-bisphosphatase. *Plant Cell Environ.* 32 811–827. 10.1111/j.1365-3040.2009.01960.x 19220782

[B48] Teow C. C. Truong V. Den McFeeters R. F. Thompson R. L. Pecota K. V. Yencho G. C. (2007). Antioxidant activities, phenolic and β-carotene contents of sweet potato genotypes with varying flesh colours. *Food Chem.* 103 829–838. 10.1016/j.foodchem.2006.09.033

[B49] Thimm O. Bläsing O. Gibon Y. Nagel A. Meyer S. Krüger P. (2004). MAPMAN: A user-driven tool to display genomics data sets onto diagrams of metabolic pathways and other biological processes. *Plant J.* 37 914–939. 10.1111/j.1365-313X.2004.02016.x 14996223

[B50] Tsai Y. C. C. Mueller-Cajar O. Saschenbrecker S. Hartl F. U. Hayer-Hartl M. (2012). Chaperonin cofactors, Cpn10 and Cpn20, of green algae and plants function as hetero-oligomeric ring complexes. *J. Biol. Chem.* 287 20471–20481. 10.1074/jbc.M112.365411 22518837 PMC3370230

[B51] Tzin V. Malitsky S. Zvi M. M. Ben Bedair M. Sumner L. Aharoni A. (2012). Expression of a bacterial feedback-insensitive 3-deoxy-d-arabino-heptulosonate 7-phosphate synthase of the shikimate pathway in *Arabidopsis* elucidates potential metabolic bottlenecks between primary and secondary metabolism. *New Phytol.* 194 430–439. 10.1111/j.1469-8137.2012.04052.x 22296303

[B52] Vogel M. O. Moore M. König K. Pecher P. Alsharafa K. Lee J. (2014). Fast retrograde signaling in response to high light involves metabolite export, MITOGEN-ACTIVATED PROTEIN KINASE6, and AP2/ERF transcription factors in *Arabidopsis*. *Plant Cell* 26 1151–1165. 10.1105/tpc.113.121061 24668746 PMC4001375

[B53] von Caemmerer S. Farquhar G. D. (1981). Some relationships between the biochemistry of photosynthesis and the gas exchange of leaves. *Planta* 153 376–387. 10.1007/BF00384257 24276943

[B54] Vranová E. Coman D. Gruissem W. (2013). Network analysis of the MVA and MEP pathways for isoprenoid synthesis. *Annu. Rev. Plant Biol.* 64 665–700. 10.1146/annurev-arplant-050312-120116 23451776

[B55] Welsch R. Zhou X. Yuan H. Álvarez D. Sun T. Schlossarek D. (2018). Clp protease and OR directly control the proteostasis of phytoene synthase, the crucial enzyme for carotenoid biosynthesis in *Arabidopsis*. *Mol. Plant* 11 149–162. 10.1016/j.molp.2017.11.003 29155321

[B56] Wright L. P. Rohwer J. M. Ghirardo A. Hammerbacher A. Ortiz-Alcaide M. Raguschke B. (2014). Deoxyxylulose 5-phosphate synthase controls flux through the methylerythritol 4-phosphate pathway in *Arabidopsis*. *Plant Physiol.* 165 1488–1504. 10.1104/pp.114.245191 24987018 PMC4119033

[B57] Xiao Y. Savchenko T. Baidoo E. E. K. Chehab W. E. Hayden D. M. Tolstikov V. (2012). Retrograde signaling by the plastidial metabolite MEcPP regulates expression of nuclear stress-response genes. *Cell* 149 1525–1535. 10.1016/j.cell.2012.04.038 22726439

[B58] Yin R. Messner B. Faus-Kessler T. Hoffmann T. Schwab W. Hajirezaei M. R. (2012). Feedback inhibition of the general phenylpropanoid and flavonol biosynthetic pathways upon a compromised flavonol-3-O-glycosylation. *J. Exp. Bot.* 63 2465–2478. 10.1093/jxb/err416 22249996 PMC3346215

[B59] Zhang H. Xie X. Kim M. S. Kornyeyev D. A. Holaday S. Paré P. W. (2008). Soil bacteria augment *Arabidopsis* photosynthesis by decreasing glucose sensing and abscisic acid levels in planta. *Plant J.* 56 264–273. 10.1111/j.1365-313X.2008.03593.x 18573192

[B60] Zhao Q. Liu C. (2018). Chloroplast chaperonin: an intricate protein folding machine for photosynthesis. *Front. Mol. Biosci.* 4:98. 10.3389/fmolb.2017.00098 29404339 PMC5780408

[B61] Zheng B. Halperin T. Hruskova-Heidingsfeldova O. Adam Z. Clarke A. K. (2002). Characterization of chloroplast Clp proteins in *Arabidopsis*: localization, tissue specificity and stress responses. *Physiol. Plant.* 114 92–101. 10.1034/j.1399-3054.2002.1140113.x 11982939

[B62] Zybailov B. Friso G. Kim J. Rudella A. Rodríguez V. R. Asakura Y. (2009). Large scale comparative proteomics of a chloroplast Clp protease mutant reveals folding stress, altered protein homeostasis, and feedback regulation of metabolism. *Mol. Cell. Proteom.* 8 1789–1810. 10.1074/mcp.M900104-MCP200 19423572 PMC2722778

