# Supplementary material for: Proteostatic Regulation of MEP and Shikimate Pathways by Redox-Activated Photosynthesis Signaling in Plants Exposed to Small Fungal Volatiles
Source: Front Plant Sci. 2021 Mar 5;12:637976. doi: 10.3389/fpls.2021.637976 (PMC7973468; doi:10.3389/fpls.2021.637976)
Supplement: Supplementary Figure 1 — External phenotypes and rosette FW of WT plants cultured in darkness in agar solidified MS medium supplemented with sucrose in the absence or continuous presence of small VCs released by adjacent A. alternata cultures for 1 week. [file Data_Sheet_1.PDF]

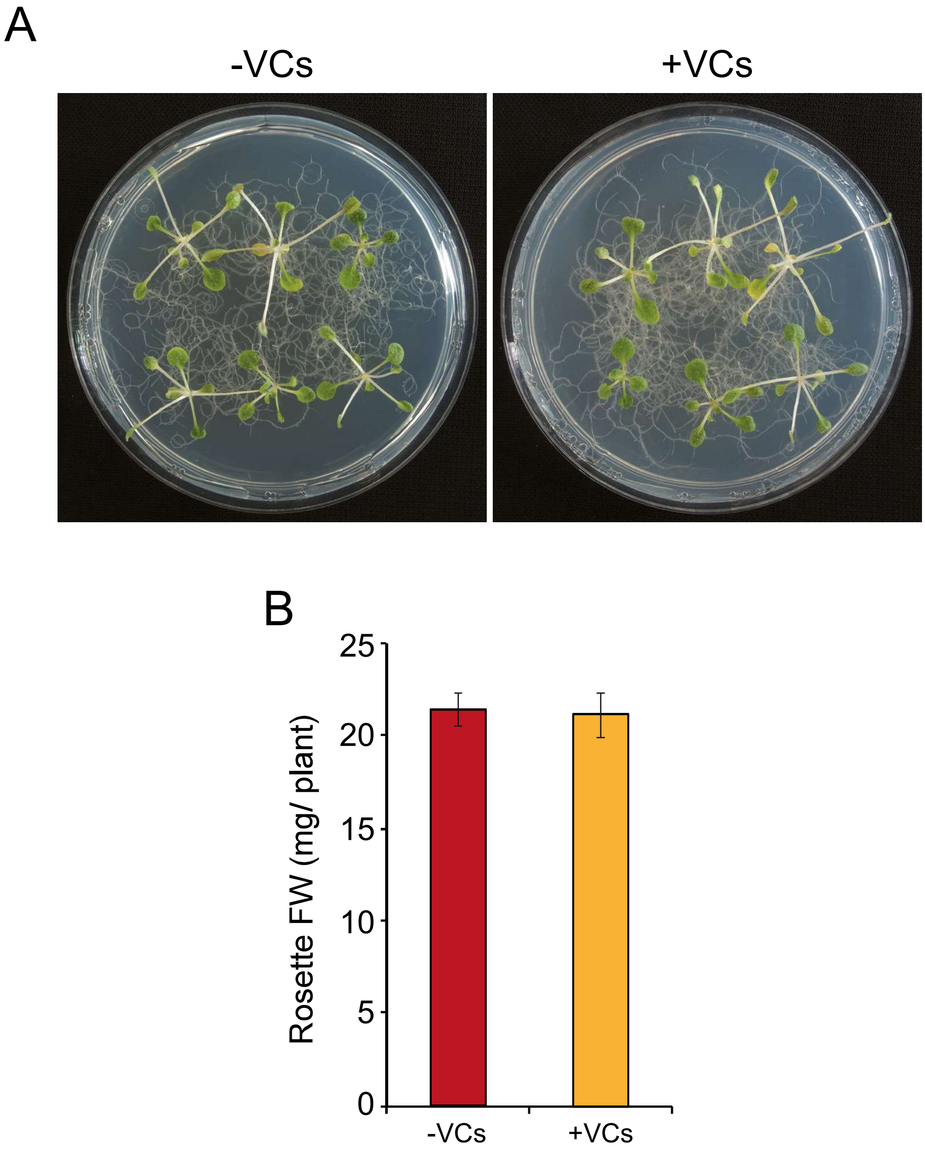

Figure S1. Light is an essential component of the response of plants to fungal VCs. (A) External phenotypes and (B) rosette FW of WT plants cultured in darkness in solid MS medium supplemented with sucrose in the absence or continuous presence of adjacent *A. alternata* cultures covered with charcoal filters for one week. Seeds were sown and plants cultured in Petri dishes under 16 h light (90 µmol photons sec^{-1} m^{-2}), 22 ºC /8 h dark, 18 ºC conditions. Fourteen days after sowing, plants were placed in the "box-in-box" co-cultivation system and cultured in darkness for one week in the absence or continuous presence of adjacent fungal cultures. Values in "B" are means ± SE for three biological replicates (each comprising a pool of 12 plants) from four independent experiments.

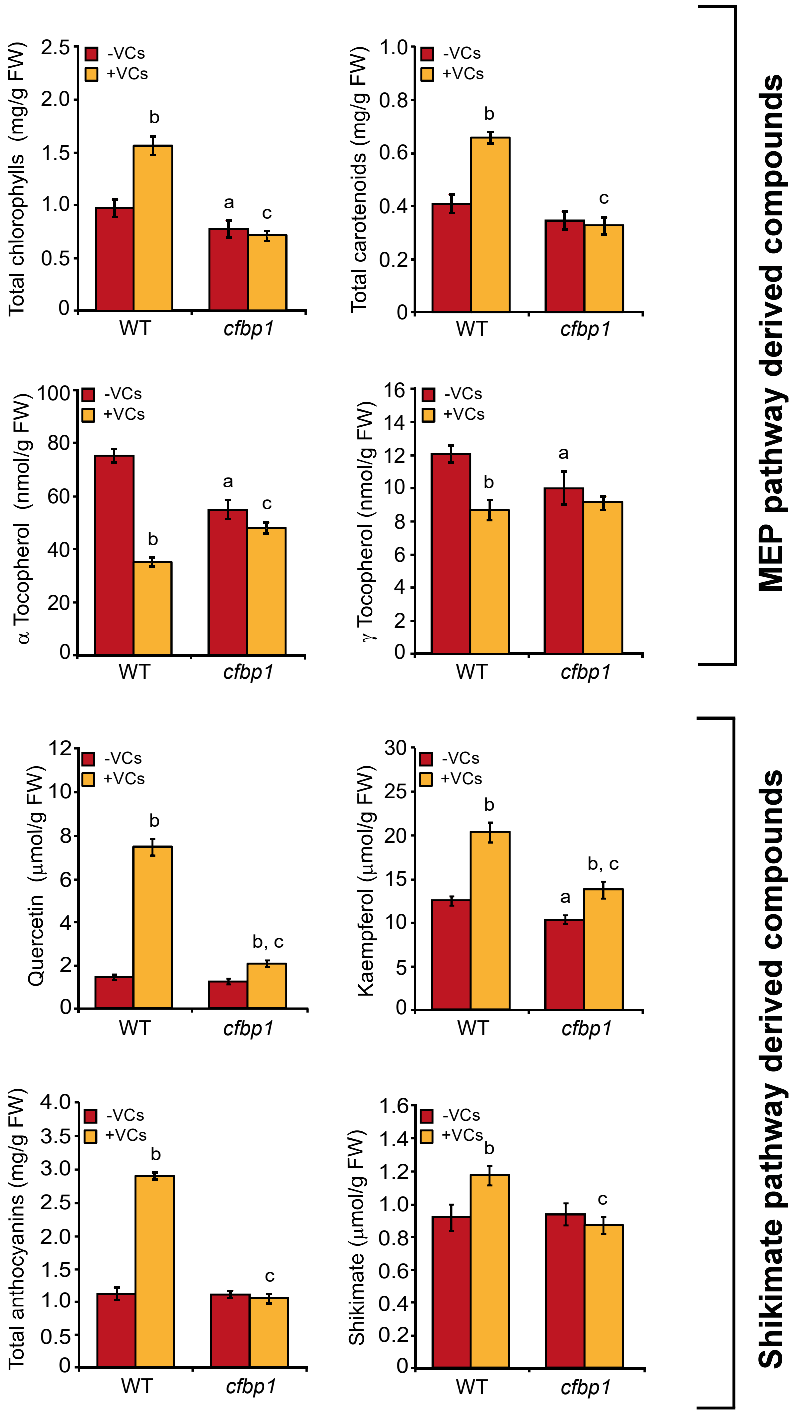

Figure S2: Fungal VCs do not alter the contents of MEP and shikimate pathways-derived compounds in leaves of *cfbp1* plants. The graphics represent the contents of MEP and shikimate pathways-derived compounds in leaves of WT and *cfbp1* plants cultured in agar solidified MS medium supplemented with sucrose in the absence or continuous presence of adjacent *A. alternata* cultures for 3 days. Values are means ± SE for three biological replicates (each comprising a pool of 12 plants) from three independent experiments. Letters 'a,' 'b,' and 'c' indicate significant differences, according to Student's t test ($p < 0.05$), between: "a" WT plants and *cfbp1* plants cultured without fungal VC treatment, "b" VC-treated and non-treated plants, and "c" VC-treated WT and mutant plants.

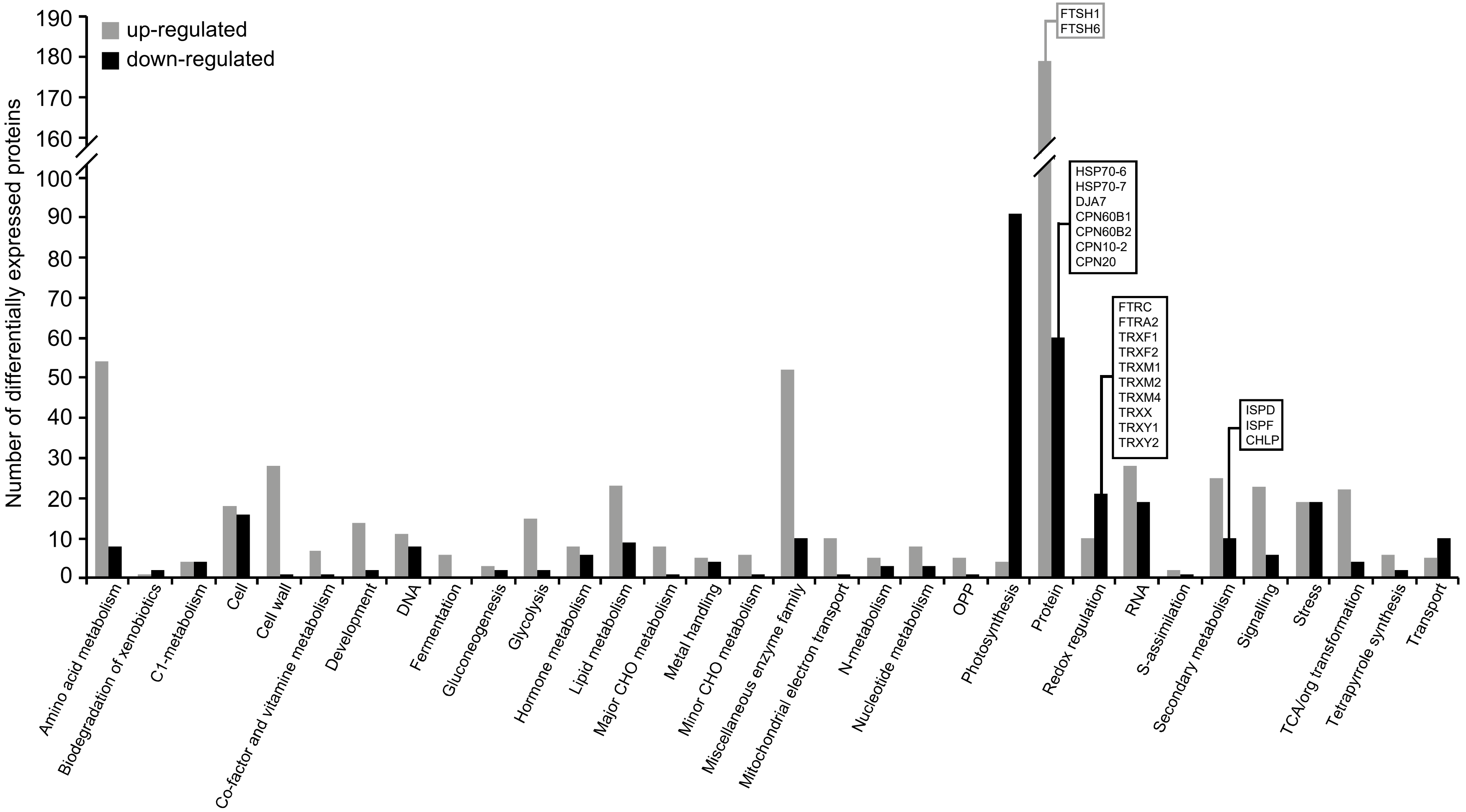

Figure S3. Knocking out cFBP1 reduces the expression of enzymes of the MEP pathways and of proteins involved in the plastidial proteostasis. The graphic represents the functional categorization of the proteins that are differentially expressed upon knocking out cFBP1. Proteins down- and up-regulated by the lack of cFBP1 expression were sorted according to the putative functional category assigned by the MapMan tool. The numbers of up- and down-regulated proteins in each categorical group are indicated by gray and black bars, respectively. Almost 1100 of the 3140 proteins identified in this proteomic study were proteins with known functions that were differentially expressed by the lack of cFBP1 with "confident" statistical significance levels (Table S8, Table S9). Among these proteins, 669 were up-regulated and 425 were down-regulated (Table S8). Note that knocking out cFBP1 reduced the expression of enzymes that control the redox status of plastidial proteins (e.g. FTRC, FTRA2, TRXF1, TRXF2, TRXM1, TRXM2, TRXM4, TRXX, TRXY1, TRXY2, BAS1 and PRXQ), MEP pathway enzymes (e.g. ISPD and ISPF) and enzymes involved in chlorophyll synthesis (e.g. CHLP). The lack of cFBP1 also enhanced the expression of 2 of the 9 plastidial ATP-dependent zinc metalloproteases (FTSH1 and FTSH6), and reduced the expression of the 2 plastidial Hsp70 chaperones (HSP70-6 and HSP70-7), one Hsp40/DnaJ family chaperone member (e.g. DJA7), 2 of the 6 plastidial chaperonins (e.g. CPN60B1 and CPN60B2) and 2 of the 3 plastidial co-chaperonins (e.g. CPN10-2 and CPN20) expressed in Arabidopsis.

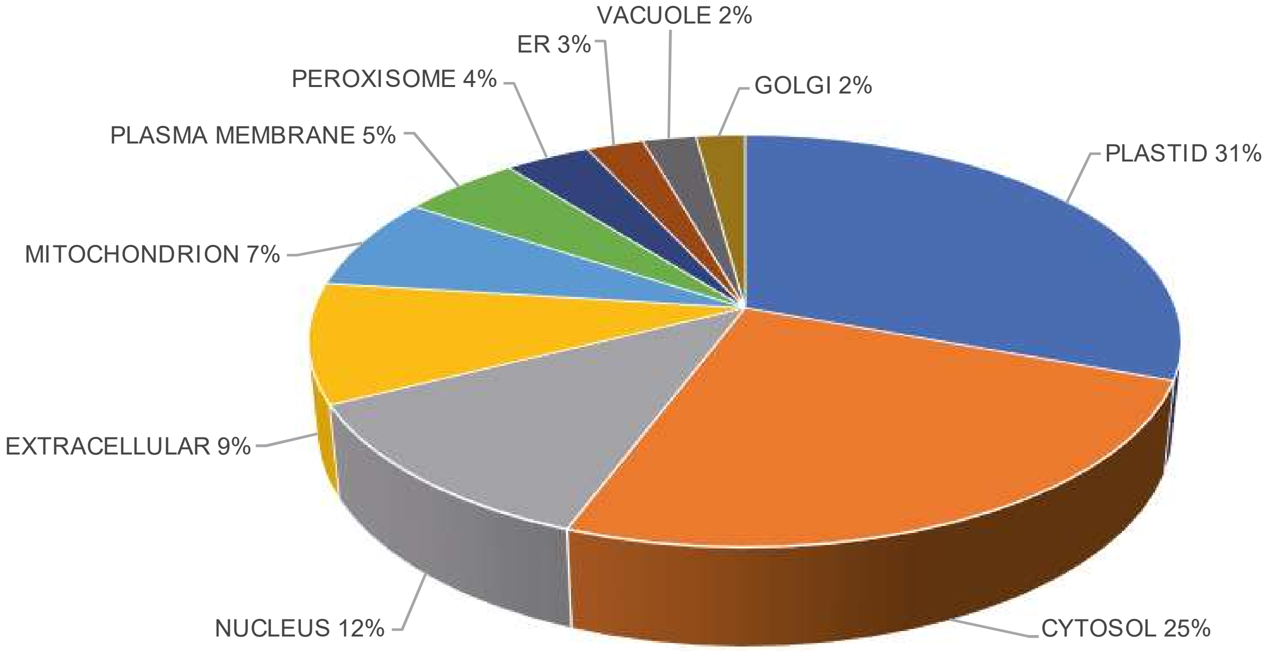

Figure S4. Categorization of fungal VC-responsive proteins according to their subcellular localizations. Data from Table S3.

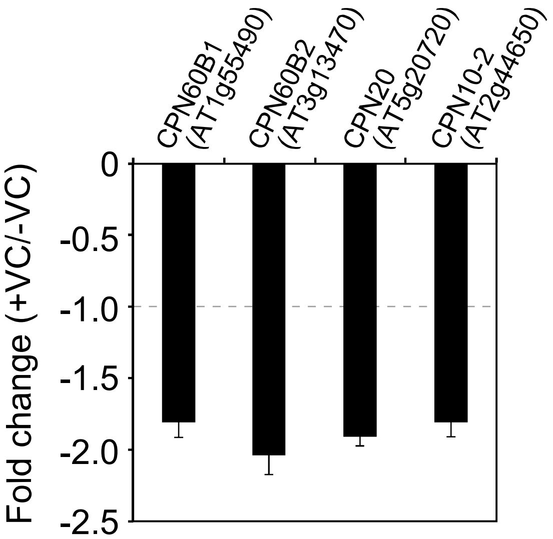

Figure S5. Fungal VCs do not enhance the expression of genes encoding plastidial chaperones in *cfbp1* plants. Relative abundance of transcripts encoding plastidial chaperones differentially expressed by fungal VCs in *cfbp1* leaves. Fold change values are differences in levels of transcripts (measured by quantitative RT-PCR) in leaves of *cfbp1* plants cultured in the presence of fungal VCs for 3 days relative to those of leaves of plants cultured in the absence of VCs. Gray dashed lines indicate the threshold of log2-fold change = 1 (2-fold change) used to identify genes significantly regulated by fungal VCs. Values are means ± SE for three biological replicates.
