# Supplementary material for: Proteostatic Regulation of MEP and Shikimate Pathways by Redox-Activated Photosynthesis Signaling in Plants Exposed to Small Fungal Volatiles
Source: Front Plant Sci. 2021 Mar 5;12:637976. doi: 10.3389/fpls.2021.637976 (PMC7973468; doi:10.3389/fpls.2021.637976)
Supplement: Supplementary Table 1 — Primers used in qRT-PCR. [file Data_Sheet_3.PDF]

**Table S1.** Primers used in qRT-PCR

| Gene | | Sequence |
|---|---|---|
| EF-1 alfa | Forward | TTCGTCTCCCACTTCAGGAT |
| At1g07940 | Reverse | GGAGCAAAGGTCACAACCAT |
| CPN60A1 | Forward | TCGTGAGGTTGCGAGTAAGA |
| At2g28000 | Reverse | CCAGAAGTGACGCTCAACAA |
| CPN60B1 | Forward | CAAGCTCGCAGATCTTGTTG |
| At1g55490 | Reverse | TTGCAACAGTCACACCATCA |
| CPN60B2 | Forward | GCAAGTATGGATCCCCAAGA |
| At3g13470 | Reverse | GCTTGCCTTACAAGCTTTGC |
| CPN60B3 | Forward | ACGGTCGCTAGAGAGGTTGA |
| At5g56500 | Reverse | CGTTGTTCCATCACCAGCTA |
| CPN20 | Forward | AGTTCCGTCGTTTGGTTGTC |
| At5g20720 | Reverse | GCCTCCTTGATCTTCACCAA |
| CPN10-2 | Forward | TAAATGGGAACCGACAAAGG |
| At2g44650 | Reverse | CACAGCTGCTTTAGGCAACA |
| ClpP5 | Forward | CGTCTCCTCAAGGAGTTTGG |
| At1g02560 | Reverse | CTTGCACCATAGGAGGTGGT |
| ClpP6 | Forward | TGGAGTGATAGAGGCGAAAAA |
| At1g11750 | Reverse | TGCGGTTACGGAATAACACA |
| ClpR3 | Forward | GCCGCCATATCTTGACATCT |
| At1g09130 | Reverse | GTCTGGTGGAGGAGTTCTCG |
