# Supplementary material for: Proteostatic Regulation of MEP and Shikimate Pathways by Redox-Activated Photosynthesis Signaling in Plants Exposed to Small Fungal Volatiles
Source: Front Plant Sci. 2021 Mar 5;12:637976. doi: 10.3389/fpls.2021.637976 (PMC7973468; doi:10.3389/fpls.2021.637976)
Supplement: Supplementary Table 2 — CK content (pmol g–1 DW) in leaves of WT and cfbp1 plants cultured in agar solidified MS medium in the absence or presence of small VCs emitted by A. alternata for 3 days. [file Data_Sheet_4.PDF]

Table S2: CK content (pmol g^{-1} DW) in leaves of WT and *cfbp1* plants cultured in solid MS medium in the absence or presence of VCs emitted by *A. alternata* for 3 days. Values represent the mean ± SE of determinations on 3 independent experiments. Asterisks indicate statistically significant difference in the VC treatments versus the controls in a paired Student's t-test (*, **, and *** correspond to P-values of 0.05 > p > 0.01, 0.01 > p > 0.001, and p < 0.001, respectively).

	**MEP pathway (plastid) derived CKs**					**MVA pathway (cytosol) derived CKs**				
		WT-VCs	WT+VCs	*cfbp1*-VCs	*cfbp1*+VCs		WT-VCs	WT+VCs	*cfbp1*-VCs	*cfbp1*+VCs
**Precursors**	**iPRMP**	48.8 ± 7.4	158 ± 10***	79.2 ± 2.0	116 ± 5***	**cZRMP**	122 ± 12	108 ± 6	111 ± 5	114 ± 7.9
	**tZRMP**	36.3 ± 4.3	232 ± 13***	38.7 ± 2.0	127 ± 8***					
	**DHZRMP**	1.7 ± 0.5	2.3 ± 0.3**	1.8 ± 0.4	1.8 ± 0.5					
	∑	86.8	392	119	245	∑	122	108	111	114
**Transport forms**	**iPR**	2.6 ± 0.3	7.0 ± 1.7***	6.8 ± 1.2	10.4 ± 1.1**	**cZR**	3.8 ± 0.4	3.1 ± 0.9	4.0 ± 0.3	4.6 ± 0.4
	**tZR**	2.8 ± 0.2	18.5 ± 2.4***	4.2 ± 0.6	15.9 ± 0.2***					
	**DHZR**	0.2 ± 0.1	0.55 ± 0.12***	0.27 ± 0.05	0.64 ± 0.13***					
	∑	5.7	26.0	11.25	26.97	∑	3.8	3.1	4.0	4.6
**Active forms**	**iP**	3.4 ± 0.2	3.8 ± 0.6	3.5 ± 0.3	3.4 ± 0.1	**cZ**	12.4 ± 1.0	5.9 ± 0.7***	9.1 ± 1.8	7.2 ± 0.3
	**tZ**	10.2 ± 0.8	18.4 ± 2.4***	7.3 ± 1.2	14.7 ± 2.4***					
	**DHZ**	--	--	--	--					
	∑	13.6	22.2	10.7	18.2	∑	12.4	5.9	9.1	7.2
**Glycosylated (inactive) forms**	**iP7G**	524 ± 23	332 ± 8***	607 ± 26	461 ± 82*	**cZ7G**	322 ± 11	109 ± 8***	368 ± 33	279 ± 19***
	**tZ7G**	284 ± 9	352 ± 15***	188 ± 14	263 ± 21***					
	**DHZ7G**	124 ± 6	63.5 ± 3.8***	111 ± 6	67 ± 6***					
	**iP9G**	43.4 ± 1.9	25.6 ± 1.8***	48.4 ± 6.5	107 ± 118	**cZ9G**	5.4 ± 0.8	1.1 ± 0.1***	6.5 ± 0.7	4.2 ± 0.2***
	**tZ9G**	91.3 ± 5.3	141 ± 10***	68.6 ± 6.1	107 ± 4***					
	**DHZ9G**	2.7 ± 0.3	2.0 ± 0.3***	2.2 ± 0.4	2.4 ± 0.1					
	**tZOG**	105.3 ± 24.1	222 ± 46**	182 ± 30	269 ± 22**	**cZOG**	33.5 ± 3.4	27.6 ± 6.0	89.6 ± 20.6	81.4 ± 7.2
	**DHZOG**	2.6 ± 0.1	2.0 ± 0.1***	2.0 ± 0.3	2.0 ± 0.1					
	**tZROG**	8.2 ± 0.3	9.0 ± 0.8	7.9 ± 0.8	11.2 ± 1.4**	**cZROG**	39.9 ± 2.1	20.7 ± 3.5***	59.8 ± 7.9	53.8 ± 10.6
	**DHZROG**	1.6 ± 0.3	1.3 ± 0.1*	1.9 ± 0.6	1.9 ± 0.3					
	∑	1,189	1,153	1,220	1,295	∑	401	158	524	418
**TOTAL**	∑	1,295	1,594	1,362	1,585	∑	539	276	649	545
