# Supplementary material for: Proteostatic Regulation of MEP and Shikimate Pathways by Redox-Activated Photosynthesis Signaling in Plants Exposed to Small Fungal Volatiles
Source: Front Plant Sci. 2021 Mar 5;12:637976. doi: 10.3389/fpls.2021.637976 (PMC7973468; doi:10.3389/fpls.2021.637976)
Supplement: Supplementary Discussion — The increase in cytokinin levels promoted by small fungal VCs is not due to an enhanced flux from the CBC to the MEP pathway. [file Data_Sheet_2.PDF]

Supplementary discussion for

# Proteostatic regulation of MEP and shikimate pathways by redox-activated photosynthesis signaling in plants exposed to fungal volatiles

**Authors:** Kinia Ameztoy[1], Ángela María Sánchez-López[1], Francisco José Muñoz[1], Abdellatif Bahaji[1], Goizeder Almagro[1], Edurne Baroja-Fernández[1], Samuel Gámez-Arcas[1], Nuria De Diego[2], Karel Doležal[2,3], Ondřej Novák[3], Ales Pěnčík[3], Adán Alpízar[4], Manuel Rodríguez-Concepción[5] and Javier Pozueta-Romero[1]

**Corresponding author:** Javier Pozueta Romero

E-mail: javier.pozueta@csic.es

Tel: (34) 948168009, Fax: (34) 948232191

**This file includes:**

Supplemental Discussion text

References

## Supplementary discussion text

### The increase in cytokinin levels promoted by small fungal VCs is not due to an enhanced flux from the CBC to the MEP pathway

We previously proposed that small fungal VC-promoted enhancement of active MEP pathway-derived CKs in leaves could be at least partly due to an enhanced metabolic flux from the CBC to the MEP pathway (Sánchez-López et al., 2016a, 2016b). However, here we found that, as in WT plants, small fungal VCs increased the levels of plastidial CKs in leaves of exposed *cfbp1* plants (**Table S2**) despite exerting weak or null stimulatory effect on photosynthesis (**Figure 2, Table 1**). As in *cfbp1* plants, fungal VCs exerted weak stimulatory effects on growth and photosynthesis in *ntrc* plants impaired in the expression of NADPH-dependent thioredoxin reductase C (NTRC), a plastidial thioredoxin that helps regulate the redox status of stromal target proteins including cFBP1 and other CBC enzymes (Ameztoy et al., 2019). Fungal VC-exposed *ntrc* plants also accumulated higher levels of MEP pathway-derived CKs than controls (Ameztoy et al., 2019). Based on these results, we propose that VC-promoted enhancement of CK content in leaves of *cfbp1* and *ntrc* mutants is not due to enhanced flux from the CBC to the MEP pathway but mainly to up-regulation of the CK biosynthetic pathway in vascular tissues of roots and subsequent transport to leaves. This hypothesis is supported by three key findings: (i) VCs do not alter the expression of CK metabolism-related enzymes in leaves (this work) but do enhance the expression of enzymes involved in CK biosynthesis in roots (García-Gómez et al., 2020), (ii) VCs do not promote photosynthesis in *cfpb1* and *ntrc* plants, and (iii) plastidial MEP pathway-derived CKs are mainly synthesized in vascular tissues of roots and transported to the aerial parts of the plant (Ko et al., 2014; Miyawaki et al., 2004).

## REFERENCES


Ameztoy, K., Baslam, M., Sánchez-López, Á. M., Muñoz, F. J., Bahaji, A., Almagro, G., et al. (2019). Plant responses to fungal volatiles involve global post-translational thiol redox proteome changes that affect photosynthesis. *Plant. Cell Environ.* 42, 2627–2644. doi:10.1111/pce.13601.

García-Gómez, P., Bahaji, A., Gámez-Arcas, S., Muñoz, F. J., Sánchez-lópez, Á. M., Almagro, G., et al. (2020). Volatiles from the fungal phytopathogen *Penicillium aurantiogriseum* modulate root metabolism and architecture through proteome resetting. *Plant. Cell Environ.* 43, 2551– 2570. doi:10.1111/pce.13817.

Ko, D., Kang, J., Kiba, T., Park, J., Kojima, M., Do, J., et al. (2014). Arabidopsis ABCG14 is essential for the root-to-shoot translocation of cytokinin. *Proc. Natl. Acad. Sci. U. S. A.* 111, 7150–5. doi:10.1073/pnas.1321519111.

Miyawaki, K., Matsumoto-Kitano, M., and Kakimoto, T. (2004). Expression of cytokinin biosynthetic isopentenyltransferase genes in Arabidopsis: Tissue specificity and regulation by auxin, cytokinin, and nitrate. *Plant J.* 37, 128–138. doi:10.1046/j.1365-313X.2003.01945.x.

Sánchez-López, Á. M., Bahaji, A., De Diego, N., Baslam, M., Li, J., Muñoz, F. J., et al. (2016a). Arabidopsis responds to Alternaria alternata volatiles by triggering plastid phosphoglucose isomerase-independent mechanisms. *Plant Physiol.* 172, 1989–2001. doi:10.1104/pp.16.00945.

Sánchez-López, Á. M., Baslam, M., De Diego, N., Muñoz, F. J., Bahaji, A., Almagro, G., et al. (2016b). Volatile compounds emitted by diverse phytopathogenic microorganisms promote plant growth and flowering through cytokinin action. *Plant Cell Environ.* 39, 2592–2608. doi:10.1111/pce.12759.
